# E-Learning for Pediatric Emergency Department Staff in Point-of-Care Electroencephalogram Interpretation: Prospective Cohort Study

**DOI:** 10.2196/69395

**Published:** 2025-08-20

**Authors:** Leopold Simma, Maurice Henri Schneeberger, Stefanie von Felten, Michelle Seiler, Georgia Ramantani, Bigna Katrin Bölsterli

**Affiliations:** 1 Emergency Department, University Children's Hospital Zurich, Lenggstrasse 30, Zurich, 8008, Switzerland, 41 44 249 49 49; 2 Children’s Research Center, University Children’s Hospital Zurich, University of Zurich, Zurich, Switzerland; 3 Master Program in Biostatistics, University of Zurich, Zurich, Switzerland; 4 Department of Biostatistics, Epidemiology, Biostatistics and Prevention Institute, University of Zurich, Zurich, Switzerland; 5 Department of Neuropediatrics, University Children's Hospital Zurich, Zurich, Switzerland; 6 Department of Pediatric Neurology, Ostschweizer Kinderspital, St. Gallen, Switzerland; 7 Child Development Center, University Children's Hospital Zurich, Zurich, Switzerland

**Keywords:** electroencephalography, medical education, seizures, status epilepticus, critical care, pediatric emergency medicine, point-of-care systems, emergency service

## Abstract

**Background:**

Status epilepticus (SE) represents a critical pediatric emergency necessitating prompt treatment and monitoring. The diagnosis of nonconvulsive SE and the monitoring of convulsive SE require electroencephalogram (EEG) recordings. The integration of simplified point-of-care EEG may improve care in pediatric emergency departments.

**Objective:**

This study aims to assess the efficacy of an electronic EEG self-learning module for improving the interpretation of normal cortical activity, artifacts, and seizure patterns in point-of-care EEG by pediatric emergency medicine (PEM) providers.

**Methods:**

This prospective cohort study was conducted in a tertiary academic pediatric emergency department and primarily targeted senior medical staff while also engaging junior medical staff and registered nurses. A novel EEG e-learning module trained participants to identify normal cortical activity, artifacts, and seizure patterns. The study comprised pretest, posttest, and 3-month retention assessments to evaluate the EEG total score as its primary outcome and basic EEG knowledge and confidence measures as secondary outcomes. Outcomes were analyzed using mixed-effects proportional odds logistic regression models.

**Results:**

Of 102 PEM providers invited, 61 individuals participated (25 senior medical staff, 15 junior medical staff, and 21 registered nurses), and 29 finished the 3-tiered study. In finishers, the EEG total score (max=12 points), indicative of accurate EEG classification, increased substantially between pretest and posttest from a median of 7 (IQR 5‐8) to 10 (IQR 7‐11) points, corresponding with an increase in the odds of achieving higher EEG total scores at the posttest (odds ratio 24.18, 95% CI 7.398-79.043, *P*<.001). At the retention test, the EEG total score remained elevated, although to a lesser extent (median 8 points [IQR 6‐9]). Similar trends were observed in secondary outcomes.

**Conclusions:**

The implementation of an e-learning EEG module improved the ability of PEM providers to interpret EEGs. This study highlights the feasibility of imparting basic EEG skills to nonexperts through targeted educational interventions. However, the sustained retention of such skills requires improvement, emphasizing the necessity for ongoing refresher training.

## Introduction

Acute central nervous system disorders are common childhood emergencies [1]. These disorders are very frequent among highly acute presentations in pediatric emergency departments (PEDs) and are the most frequent type of presentation in pediatric resuscitation bays [2-4]. Status epilepticus (SE) is a paramount medical emergency in pediatric neurology. Early identification of SE in PEDs is crucial for prompt initiation of treatment and thus for improving patient outcomes [5]. However, nonconvulsive SE (NCSE) often eludes detection, particularly in patients with altered mental status (AMS) or seemingly controlled convulsive SE [6,7]. Standard electroencephalogram (EEG) is the gold standard for monitoring SE treatment and detecting NCSE due to its high sensitivity in identifying electrographic-only seizure activity [8].

Although electroencephalograms (EEGs) in PEDs provide valuable diagnostic information and aid in decision-making [9,10], immediate access to standard EEG may be challenging in many settings [10]. Low standard EEG availability poses a challenge, both during and after standard working hours, because standard EEG requires significant time, equipment, and specialized personnel [11]. A simplified, reduced-lead EEG, also termed point-of-care EEG (pocEEG), has emerged as a feasible option [12] to aid diagnostics [13-15] and facilitate the management of AMS, the treatment and monitoring of SE, and the detection of NCSE [15,16]. However, the interpretation of pocEEG by nonexpert PED staff in the absence of or with delayed access to EEG experts, such as pediatric neurologists, is challenging, undermining the applicability of this novel approach in the PED setting [17].

Previous research has explored efforts to train nonexperts in EEG interpretation in both adult and neonatal and pediatric acute care settings [18,19], with most studies focusing on processed EEG, such as amplitude integrated EEG or continuous EEG in intensive care units [18]. Yet, only one study has investigated the acquisition of standard EEG interpretation skills in adult emergency department (ED) physicians [20]. To date, data are lacking on the feasibility and efficacy of teaching basic EEG skills to pediatric emergency medicine (PEM) providers, even though here this approach could significantly improve patient care by facilitating AMS evaluation and expediting NCSE diagnosis and treatment [17,21]. To address this gap, we designed a novel pocEEG e-learning module to transfer basic EEG knowledge of montages, EEG signal generation, and EEG reading and interpretation skills to PEM providers. The module focuses on identifying normal cortical activity, artifacts, and seizure patterns. These 3 domains are important for distinguishing between true neurological events and artifacts, and thus for identifying and managing seizures.

Therefore, the primary aim of this study was to determine the impact of our EEG e-learning module on EEG skill acquisition by nonexpert PED staff through a 3-tiered longitudinal assessment. Secondary aims were to assess whether training led to measurable improvement in recognizing specific EEG patterns, self-assessment, and confidence among PEM providers over time.

## Methods

### Study Design and Setting

This study was conducted at the tertiary academic PED of the University Children’s Hospital Zurich, Switzerland, which treats 50,000 patients annually. The department is staffed by diverse professional groups, including senior medical staff (SMS) such as PEM attendings and PEM fellows, junior medical staff (JMS) such as pediatric residents and pediatric surgery residents, registered nurses (RNs), and medical practice assistants.

In this prospective cohort study, we tested an e-learning module as an intervention primarily targeting the SMS, who have the final responsibility for patient evaluations. However, all PEM providers were invited to participate in a 3-visit program consisting of a baseline pretest followed by a posttest and a 3-month retention test.

### Study Population

For enrollment, 102 PEM providers were invited via email using the REDCap (Research Electronic Data Capture; Vanderbilt University) survey tool [22]. We extended invitations for anonymous participation in this e-learning module to all SMS, JMS, and RNs within our PED. Our primary goal was to actively engage the majority of SMS while also providing access to EEG training and promoting the use of pocEEG in the PED by JMS and RNs. After completion of the pretest, participants were provided with a passcode to access the learning module. One hour after pretest completion, participants were provided with another survey link and prompted to complete the posttest by entering a passcode provided on module completion. The retention test was automatically scheduled 3 months following posttest completion. Up to 4 automated reminders were sent to encourage participation between 88 and 120 days after pretest. Pretest participants who failed to complete the learning module were invited to join a control group by completing the 3-month retention test.

### Study Protocol

In a collaborative effort, a PEM expert and a neurophysiologist (LS and BKB) devised an innovative e-learning module aimed at facilitating the use and interpretation of pocEEG. Drawing on insights from a previous adult study [20], this module incorporates audio-guided PowerPoint (Microsoft Corp) presentations. The content covers fundamental EEG principles, including electrode placement according to the 10‐20 system, a standardized method of electrode placement, and insights into standard EEG and pocEEG findings. The module focuses on identifying normal EEG waveforms, artifacts, and seizure patterns in accordance with the American Clinical Neurophysiology Society 2021 guidelines [23].

The main segment of the module, spanning about 60 minutes, is dedicated to two primary objectives: (1) providing a systematic framework for assessing cortical activity by familiarizing participants with normal EEG waveforms, and (2) educating participants about critical EEG findings such as seizure patterns in pocEEGs. Additionally, a 20-minute chapter offers technical instructions for pocEEG covering electrode placement, EEG, and optionally video recording, and troubleshooting techniques. Although this technical knowledge is not tested, it enhances participants’ understanding of pocEEG procedures.

### Measurements and Test Materials

**Figure 1.**
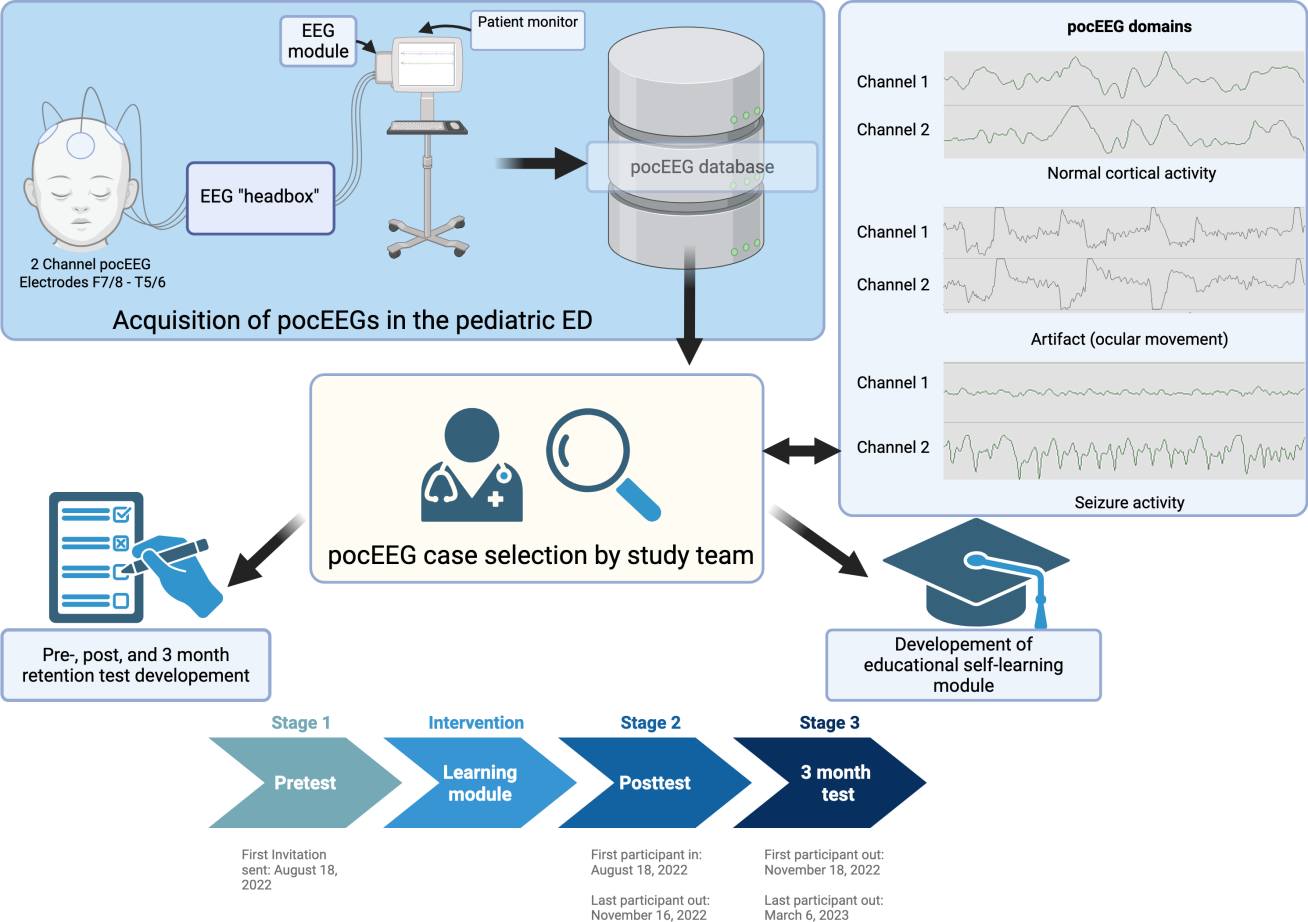
pocEEG acquisition, learning module, and test development (created with BioRender.com). EEG: electroencephalogram; pocEEG: point-of-care electroencephalogram.

Participants were assessed on their ability to distinguish normal cortical activity, artifacts, and seizure patterns by reviewing 12 anonymized pocEEGs. These pocEEGs were acquired via patient monitors from patients presenting to the PED during the introduction phase of this modality [24] and consisted of 2-channel pocEEG recordings (channels F7/8 and T5/6; 10‐20 system) as described elsewhere [24]. These recordings were processed with Vitalrecorder (version 1.8.16.4) [25], and educationally valuable EEGs were carefully selected from the pocEEG database by PEM and neurophysiology experts (LS and BKB; Figure 1).

Each test session included basic demographic data on the participants’ profession, seniority, and post board certification experience; a self-assessment of confidence; and basic EEG knowledge. The 12 EEG interpretation tasks comprised 7 pocEEG trace snapshots (10 s duration) and 5 pocEEGs with video segments lasting up to 60 seconds each. Each of the 3 domains, normal cortical activity, artifacts, and seizure patterns, was assessed with 4 pocEEGs. EEG interpretation knowledge was tested with single-choice questions, each with 5 answer options. Each answer included a statement about background symmetry, dominant activity, and whether the pocEEG included an artifact or indicated seizure activity. Additionally, participants were asked to disclose which of their answers they had guessed [26]. To minimize bias, question sequences varied across all 3 tests. A pediatric neurology fellow who was not involved in the study piloted the test material. Feedback was used to adjust item difficulty and ensure technical feasibility. Study data were collected and managed using the REDCap electronic data capture tool hosted at the University Children’s Hospital Zurich [22]. We report this survey data in accordance with the CHERRIES (Checklist for Reporting Results of Internet E-Surveys) guidelines [27], and a detailed CHERRIES checklist is provided in Checklist 1.

### Outcome Measures

We assessed both the theoretical knowledge and practical skills of participants in pocEEG interpretation through a series of 3 tests, which yielded these outcomes:

- Primary Outcome
  - EEG total score: This score is calculated from the accurate classification of 12 pocEEG recordings into categories of normal cortical activity, artifacts, or seizure patterns. Scores range from 0 (no correct answers) to 12 (all correctly answered).
- Secondary Outcomes
  - EEG basic knowledge score: This score reflects the accuracy of answers to 5 basic EEG knowledge questions (single choice, each with 4 answer options) evaluated across 3 tests. Scores range from 0 (no correct answer) to 5 (all answered correctly).
  - Classification accuracy per domain: The 12 EEG recordings are categorized into 3 domains: normal activity, artifacts, and seizure patterns, each represented by 4 EEGs. The classification of each EEG recording is evaluated with a binary outcome (correct/incorrect).
  - Assurance level: This includes the total number of answers guessed per test, ranging from 0 (no guesses) to 12 (all guessed), and the number of correct, deliberate answers per test, ranging from 0 to 12.
  - Self-assessment and confidence measures: Five items measured on a Likert scale from 1 (completely agree) to 5 (completely disagree).
  - Estimation of an intervention effect: To measure the effect of the e-learning module, we compare the EEG total scores of finishers and the control group in the 3-month retention test.

### Statistical Analysis

Descriptive statistics of study participants were tabulated overall, by professional group, and by group depending on test completion (see below). We report frequency and percentage for categorical variables.

The ordinal primary outcome EEG total score was analyzed by mixed-effects proportional odds logistic regression (PolrME) and mixed-effects enhanced proportional odds logistic regression (ePolrME) with the PolrME and ColrME functions in the R package tramME [28]. For simplicity, we report only the ePolrME results. To account for repeated measurements, random intercepts per study participant were included in the models. To investigate changes in the EEG total score between tests in participants who completed all tests, models were fitted with test (pre-, post-, and 3 mo retention) and professional group (SMS, JMS, and RNs) as explanatory variables. The analysis primarily focused on SMS, the largest professional group. To test whether changes in EEG total score across all tests differed between professional groups, we included an interaction between test and professional group in the models and used likelihood ratio tests to assess whether interaction terms improved model fit.

Similar models were fitted for the secondary outcome EEG basic knowledge score. To investigate the effect of completing the e-learning module as an intervention on EEG total score, we fitted an ordinary proportional odds logistic regression model to participants who completed all components of the study and to a control group who only completed the pretest and retention test without engaging with the e-learning module or posttest. The EEG total score at the pretest and completion of the e-learning module (yes/no) was used as explanatory variables.

The PolrME models (and proportional odds logistic regression model) estimate odds ratios (ORs) for falling into a higher EEG total score category, comparing a given level of the explanatory variables to the reference group (eg, posttest vs pretest or e-learning module completed vs not completed) for a given study participant. Similarly, ePolrME models estimate ORs for higher EEG total scores. The interpretation remains consistent when the models are applied to the secondary outcome, EEG basic knowledge. To assess differences in recognizing seizure patterns, artifacts, and normal cortical activity, a binomial general linear mixed effects model with logit link function was fitted to the correct EEG rating (binary outcome, 1=correct, 0=false). The data set was prepared to contain a row for each rating of an EEG by a participant (12 EEG recordings in each test and participant). The model included a random intercept for test nested within study participant and the type of EEG (normal cortical activity, artifact, or seizure pattern) and test as explanatory variables, and estimated ORs for correct rating of EEG recordings. All analyses were performed using R (version 4.1.2; R Foundation for Statistical Computing) [29].

### Ethical Considerations

This study involves human participants but does not fall within the scope of the Swiss Human Research Act, and the responsible ethics committee (Cantonal Ethics Committee, Zürich, Switzerland) exempted this study (Req-2024‐00833). All participants were volunteers and gave electronic consent to participate in the study.

## Results

### Overview

**Figure 2.**
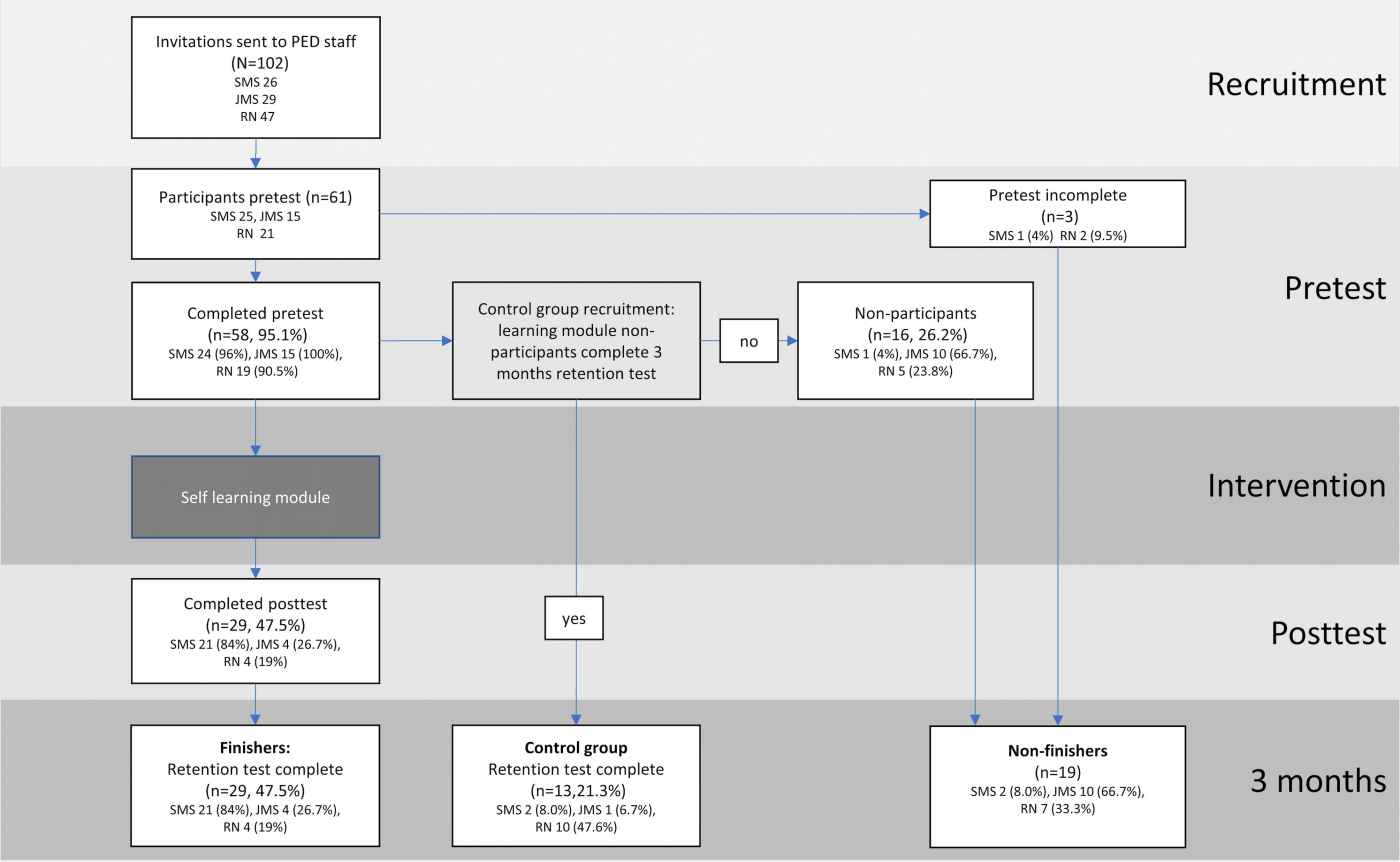
Flowchart of the study’s stages from recruitment to pretest, intervention, posttest, and study conclusion. Percentages are calculated relative to the initial participant pool who took the pretest (n=61). Subgroup percentages always refer to the respective subgroup sample. JMS: junior medical staff (residents); RN: registered nurse; SMS: senior medical staff (attendings/consultants; fellows).

In all, 102 PEM providers were invited to participate in the study, and 61 consented to take the pretest (Figure 2), including 25 SMS, 15 JMS, and 21 RNs (Table 1). A total of 53 (86.9%) participants were female, 25 (41.0%) participants were SMS, 15 (24.6%) participants were JMS, and 21 (34.4%) participants were RNs. Participants had minimal or no prior experience in implementing and interpreting EEGs.

**Table 1.** Participant characteristics.

	Overall (n=61)	Control group (n=13)	Finisher (n=29)	Nonfinisher (n=19)
Sex, n (%)				
Female	53 (86.9)	11 (84.6)	24 (82.8)	18 (94.7)
Male	8 (13.1)	2 (15.4)	5 (17.2)	1 (5.3)
Professional group, n (%)				
Senior medical staff	25 (41.0)	2 (15.4)	21 (72.4)	2 (10.5)
Junior medical staff	15 (24.6)	1 (7.7)	4 (13.8)	10 (52.6)
Registered nurses	21 (34.4)	10 (76.9)	4 (13.8)	7 (36.8)
ED^a^ experience, n (%)				
0-5 y	15 (24.6)	4 (30.8)	9 (31.0)	2 (10.5)
5-10 y	11 (18.0)	4 (30.8)	5 (17.2)	2 (10.5)
10-15 y	10 (16.4)	1 (7.7)	9 (31.0)	0 (0.0)
+15 y	10 (16.4)	3 (23.1)	2 (6.9)	5 (26.3)
JMS^b^	15 (24.6)	1 (7.7)	4 (13.8)	10 (52.6)
Prior EEG^c^ experience, n (%)				
No (no prior experience)	18 (29.5)	5 (38.5)	6 (20.7)	7 (36.8)
No (passive knowledge)	18 (29.5)	4 (30.8)	10 (34.5)	4 (21.1)
Yes (neonatal EEG^c^ [aEEG^d^])	19 (31.1)	3 (23.1)	9 (31.0)	7 (36.8)
Yes (practical experience)	6 (9.8)	1 (7.7)	4 (13.8)	1 (5.3)
Yes (proficient)	0 (0.0)	0 (0.0)	0 (0.0)	0 (0.0)

a ED: emergency department.

b JMS: junior medical staff.

c EEG: electroencephalogram.

d aEEG: amplitude integrated electroencephalogram.

Of the 61 participants who took the pretest, 58 completed it and subsequently gained access to the e-learning module. Among these, 29 completed the e-learning module, posttest, and retention test and were designated as finishers (Table 1). Participants who only completed the pretest (n=29) and did not interact with the e-learning module formed a control group and were invited to take the 3-month test, resulting in 13 controls with a pretest and a 3-month test. The group of nonfinishers (n=19) consisted of 3/61 without and 16/61 with a complete pretest. The finisher rate among SMS, the primary focus group of our e-learning module, was 84% (21/25), while it was 26.7% (4/15) in JMS, and 19.0% (4/21) in RNs. The distribution of the various participants is shown in Figure 2.

### Primary Outcome EEG Total Score

**Figure 3.**
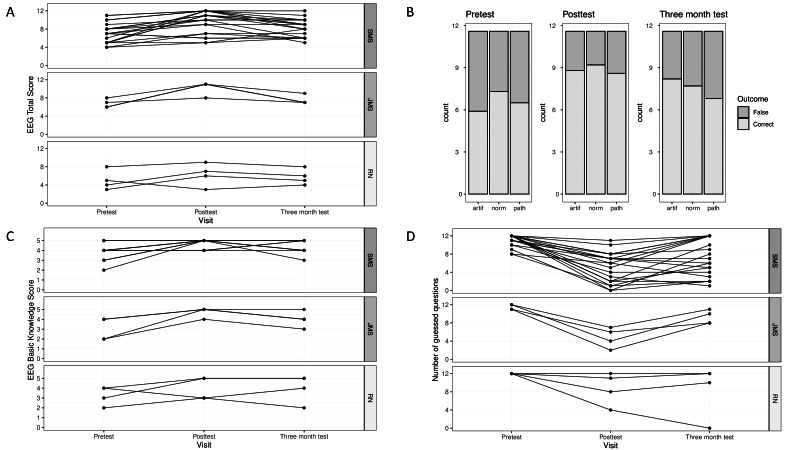
(A) Line plot for the progression of EEG total scores over pretest, posttest, and 3-month retention test of finishers: top panel senior medical staff (SMS, n=21), middle panel junior medical staff (JMS, n=4), and bottom panel registered nurses (RN, n=4). (B) Barplots for number of correct answers on pretest, posttest, and 3-month retention tests and per EEG domain artifacts (Artif), normal cortical activity (Norm), and seizure patterns (Path). (C) Lineplot showing the progression of scores in basic EEG knowledge; staff groups are shown in 3 separate panels. (D) Lineplot for progression of nonguessed correct responses (EEG total score) across the 3 tests with top panel SMS, middle panel JMS, and bottom panel RNs. EEG: electroencephalogram; JMS: junior medical staff; RN: registered nurse; SMS: senior medical staff.

The primary outcome, EEG total score, increased considerably on average following completion of the e-learning module among all finishers. However, it subsequently decreased between the posttest and the retention test, although it remained higher than at the pretest (Figures 3A and 3B, Table 2, Figure S1 in Multimedia Appendix 1).

**Table 2.** Primary outcome electroencephalogram (EEG) total scores of finishers, finisher subgroups, control group, and nonfinishers at all 3 tests (maximum score=12). Results are summarized as median and IQR (1st and 3rd quartile).

	Finishers total (n=29)	Finisher SMS^a^ (n=21)	Finisher JMS^b^ (n=4)	Finisher RNs^c^ (n=4)	Control group (n=13)	Nonfinisher (n=19)
EEG (total scores pretest), median (IQR)	7.00 (5.00-8.00)	7.00 (5.00-8.00)	6.50 (6.00-7.25)	4.50 (3.75-5.75)	5.00 (3.00-7.00)	6.00 (5.00-7.00)
EEG (total scores posttest), median (IQR)	10.00 (7.00-11.00)	10.00 (7.00-12.00)	11.00 (10.25-11.00)	6.50 (5.25-7.50)	0.00 (0.00-0.00)	0.00 (0.00-0.00)
EEG (total scores 3m^d^ test), median (IQR)	8.00 (6.00-9.00)	9.00 (6.00-10.00)	7.00 (7.00-7.50)	5.50 (4.75-6.50)	4.00 (3.00-7.00)	0.00 (0.00-0.00)

a SMS: senior medical staff.

b JMS: junior medical staff.

c RN: registered nurse.

d 3m: 3 months.

Results from the ePOLR model provide evidence for an increase in the odds of achieving higher EEG total scores at the posttest compared with the pretest (OR 24.18, 95% Wald CI 7.398-79.043; *P*<.001). Similarly, the odds of achieving higher EEG total scores at the retention test compared with the pretest increased by a factor of 3.7 (OR 3.7, 95% Wald CI 1.406-9.741; *P*=.008). Furthermore, differences emerged in EEG total scores among professional groups, with SMS achieving the highest scores, followed by JMS and RNs (OR compared with SMS of 0.79, 95% Wald CI 0.08-8.06; *P*=.84; and 0.03, 95% Wald CI 0.00-0.33; *P*=.005, respectively; Table 3).

**Table 3.** Odds ratio (OR) estimates with 95% Wald CIs from the enhanced proportional odds logistic regression model on the primary outcome, electroencephalogram total score. The model included 87 observations from 29 participants.

	OR (95% CI)	*P* value
JMS^a^ versus SMS^c^	0.79 (0.08-8.06)	.84
RNs^b^ versus SMS	0.03 (0.00-0.33)	.005
Posttest versus pretest	24.18 (7.40-79.05)	<.001
3 mo retention test versus pretest	3.70 (1.41-9.74)	.008

a JMS: junior medical staff.

b RN: registered nurse.

c SMS: senior medical staff.

### Secondary Outcomes

Among SMS, we observed that EEG basic knowledge generally increased after the intervention, with a less pronounced decline at the retention test than at the EEG total score. This pattern was also seen in JMS and RNs, albeit with some individual variations (Figure 3C). In the ePolrME model, SMS odds for a higher score in basic EEG knowledge after the module increased significantly for the posttest (OR 32.89, 95% Wald CI 7.137-151.569; *P*<.001) and for the retention test (OR 12.96, 95% Wald CI 3.421-49.122; *P*<.001). JMS and RNs showed a decrease in odds for a higher score compared with SMS (OR 0.09 Wald CI 0.01-0.79, *P*=.03; and OR 0.06, Wald CI 0.01-0.52, *P*=.01; respectively).

In the secondary outcome analysis of EEG domains, the number of correct answers for normal cortical activity (norm), artifacts (artif), and seizure patterns (path) is shown in Figure 3B. Overall, the number of correct answers increased markedly in the posttest and remained higher in the retention test compared with baseline (Table 4). We observed minor variations among the 3 domains across these tests.

**Table 4.** Odds ratio (OR) estimates with 95% Wald CIs from the binomial generalized linear mixed-effects model on classification accuracy (binary outcome for correctness of answer). The model was fitted with a logit link function and a random intercept for visit nested within participant and included 1044 observations from 29 participants.

	OR (95% CI)	*P* value
Intercept	1.16 (0.78-1.73)	.47
Artifact versus pathological	1.15 (0.83-1.61)	.40
Normal cortical activity versus pathological	1.40 (1.00-1.96)	.049
Posttest versus pretest	2.75 (1.95-3.87)	<.001
Three-month retention test versus pretest	1.50 (1.09-2.07)	.014

To assess the participants’ assurance level, we analyzed the guess rates for 12 EEG recording questions across professional groups (Figure 3D). The pretest revealed high guess rates across all groups, with SMS guessing between 8 and 12 questions, and many participants guessing all. Completion of the e-learning module was followed by a notable reduction in guess rates across all groups, with some senior staff not guessing any. However, at the 3-month retention test, guess rates increased again for all groups compared with posttest.

We evaluated the confidence levels of finishers in their interpretation of pocEEG by focusing on overall pocEEG skills, ability to detect pocEEG signals correctly, knowledge about potential artifacts, and proficiency in identifying artifacts on a Likert scale (Figures S2-S6 in Multimedia Appendix 1). During the pretest, responses regarding overall pocEEG skills varied widely across all groups, indicating a significant disparity in confidence levels. However, an improvement in confidence was observed across all occupational groups during the posttest. This increased confidence largely remained at the retention test, albeit with some variations among groups. Notably, SMS consistently exhibited higher confidence in overall skills throughout the study compared with JMS and RNs. Confidence in detecting pocEEG signals varied widely across occupational groups during the pretest but improved significantly after the completion of the e-learning module. This increased confidence remained high in the retention test, although JMS and RNs showed lower confidence levels than SMS. Likewise, confidence in identifying EEG artifacts was initially low across all occupational groups but improved notably after the e-learning module. This increased confidence generally remained in the retention test, with some variations observed among groups. Throughout the study, confidence in interpreting pocEEG recordings remained relatively low across all occupational groups, with only slight increases observed in certain subgroups and a general trend of continued uncertainty or disagreement.

### Effect of the E-Learning Module: Finishers Versus Control Group

Comparing finishers with the control group revealed that the e-learning module led to increased EEG total scores at the retention test after 3 months.

The odds of achieving higher EEG total scores in the retention test were significantly increased by a factor of 9 for the finishers compared with the controls (OR 9.075, 95% CI 2.181-37.763; *P*=.002). Furthermore, adjusting for initial EEG knowledge at pretest was important because participants with better pretest performance also performed better at the 3-month retention test (OR 2.053, 95% CI 1.505-2.800; *P*<.001).

## Discussion

### Principal Findings

This study evaluated the effectiveness of a novel e-learning EEG module in improving pocEEG interpretation skills among SMS, JMS, and RNs in the PED. Our findings indicate a notable improvement in EEG knowledge and interpretation skills across all participant groups following the module, which was particularly evident in the EEG total score, our primary outcome. These results are consistent with the literature emphasizing the importance of targeted educational interventions for nonexperts in interpreting EEGs in critical care settings [18]. To our knowledge, this study represents the first attempt to evaluate knowledge transfer of basic EEG skills to EEG nonexperts in the PED and the first investigation into training for reduced lead EEG and pocEEG skills. A recent review has also identified this topic as a research priority [17].

The primary outcome, the EEG total score, showed a substantial increase in correct classifications of EEG recordings immediately after module completion and, to a lesser extent, at the 3-month follow-up. This finding is encouraging because it demonstrates both the immediate impact and the lasting effect of the educational module, albeit with a decline over time. The increase in EEG total scores was particularly pronounced among the primary target group, SMS.

However, due to our limited sample size for JMS and RNs, we did not assess whether the increase in EEG total scores or EEG knowledge in general differed from SMS. Overall, finishers were significantly more likely to achieve higher EEG total scores at the 3-month retention test compared with the control group, with the caveat that the control group had a divergent composition (Figure 2 and Table 1).

The finisher rate among SMS was 84%, considerably higher than among JMS and RNs. This discrepancy can be attributed to several factors: JMS typically rotate through our PED for 3 to 6 months, and both JMS and most RNs lack nonclinical time in the PED and remote access to the hospital’s computer system, thus preventing their participation from home. This underscores the need for tailored educational approaches that consider the specific needs, time constraints, and baseline competencies of various health care professionals.

Seizure pattern recognition appeared to be a slightly more challenging task for participants (Figure 3B) in our set of test EEGs. Extrapolating from our experience with point-of-care ultrasound, we had previously hypothesized that identifying seizure patterns would be more straightforward than discerning normal activity [30] or artifacts, but findings may differ with a larger set of EEGs.

Nevertheless, the improvement observed in identifying artifacts and normal cortical activity is encouraging and indicates a fundamental enhancement in EEG interpretation skills. The 3 domains of normal signal, artifact, and seizure pattern form the foundational pillars of continuous EEG teaching [31]. They are crucial for improving neurocritical care in the PED, where timely and accurate EEG interpretation can significantly impact patient management and outcomes.

Our additional observations, focusing on self-assessment and confidence measures, indicated a notable increase in confidence levels among participants. The initial high rate of guesses, which decreased significantly post training, highlights the initial uncertainty among staff members in interpreting EEGs. However, the resurgence in guess rates at the 3-month follow-up suggests that periodic refresher modules or ongoing training might be necessary to maintain confidence and competence levels. This retention challenge, as with any critical care skill, is particularly pronounced in ED settings [32,33].

A recent review by Taran et al [18] outlined numerous studies conducted in critical care settings involving nurses and physicians as participants and focusing on EEG educational initiatives. These studies often evaluated the use of processed EEGs, such as amplitude-integrated EEGs, with raw EEGs being less common [18]. Only one study in the adult ED context improved the abilities of emergency physicians to identify seizure patterns; that study used a learning module with full montage standard EEG examples [20]. Studies investigating long-term retention of EEG knowledge reported better scores [19], predominantly in intensive care units, where trainees had frequent exposure to continuous EEG.

One of the key strengths of our study is its high participation rate among SMS, our primary focus group. The 3-tiered longitudinal assessment helps to gauge knowledge retention over time and assess the long-term impact of the intervention. The involvement of diverse occupational groups in the study also provides a broader understanding of educational needs across different levels of PEM providers. By assessing guess rates, we also examined participants’ confidence in their answers.

However, our study also has limitations. Its participants are all from a single pediatric academic tertiary center, which limits generalizability. The sample size of finishers other than SMS was small and limited the statistical analysis of differences between professional groups. In addition, the disparity in subgroup sizes contributed to substantial uncertainty in OR estimates, particularly for the EEG total score and EEG basic knowledge score. The reliance on self-assessment measures for confidence may introduce bias and may be influenced by local cultural factors. The composition of the control group was suboptimal due to the finite availability of study participants at the study site, which precluded the recruitment of matched controls. Selective participation and initial participant attrition limit the generalizability of our findings to broader interdisciplinary teams. In addition, the test instrument in this study was tailored for the local educational context and was not formally validated. It is unclear whether any participants engaged in interpreting pocEEGs on clinical shifts during the interval prior to the retention test, which could have influenced their knowledge retention. Finally, the decline in knowledge retention over time suggests the need for ongoing training and support.

### Conclusions

Our study demonstrates that a targeted educational module can improve EEG knowledge and interpretation skills among PED staff. This finding has important implications for enhancing neurocritical care in pediatric emergency settings, where rapid and accurate diagnosis is crucial for patient outcomes. Moreover, the decline in skill retention over time underscores the critical need for continuous education and ongoing training to ensure that these vital skills are maintained.

## Supplementary material

10.2196/69395 Multimedia Appendix 1 Additional figures.

10.2196/69395 Multimedia Appendix 2 Dataset.

10.2196/69395 Checklist 1 Checklist for Reporting Results of Internet E-Surveys (CHERRIES).

## Abbreviations

AMS: altered mental status
CHERRIES: Checklist for Reporting Results of Internet E-Surveys
ED: emergency department
EEG: electroencephalogram
ePolrME: mixed-effects enhanced proportional odds logistic regression
JMS: junior medical staff
NCSE: nonconvulsive status epilepticus
OR: odds ratio
PED: pediatric emergency department
PEM: pediatric emergency medicine
pocEEG: point-of-care electroencephalogram
PolrME: mixed-effects proportional odds logistic regression
REDCap: Research Electronic Data Capture
RN: registered nurse
SE: status epilepticus
SMS: senior medical staff

## References

[1] Pallin DJ, Goldstein JN, Moussally JS, Pelletier AJ, Green AR, Camargo CA (2008). Seizure visits in US emergency departments: epidemiology and potential disparities in care. Int J Emerg Med.

[2] Chavez H, Garcia CT, Sakers C, Darko R, Hannan J (2018). Epidemiology of the critically Ill child in the resuscitation bay. Pediatr Emerg Care.

[3] Lutz N, Vandermensbrugghe NG, Dolci M, Amiet V, Racine L, Carron PN (2014). Pediatric emergencies admitted in the resuscitation room of a Swiss university hospital. Pediatr Emerg Care.

[4] Simma L, Stocker M, Lehner M, Wehrli L, Righini-Grunder F (2021). Critically Ill children in a Swiss pediatric emergency department with an interdisciplinary approach: a prospective cohort study. Front Pediatr.

[5] Gaínza-Lein M, Fernández IS, Ulate-Campos A, Loddenkemper T, Ostendorf AP (2019). Timing in the treatment of status epilepticus: from basics to the clinic. Seizure.

[6] Sánchez Fernández I, Sansevere AJ, Guerriero RM (2017). Time to electroencephalography is independently associated with outcome in critically ill neonates and children. Epilepsia.

[7] Fung FW, Abend NS (2020). EEG monitoring after convulsive status epilepticus. J Clin Neurophysiol.

[8] Tay SKH, Hirsch LJ, Leary L, Jette N, Wittman J, Akman CI (2006). Nonconvulsive status epilepticus in children: clinical and EEG characteristics. Epilepsia.

[9] Fernández IS, Loddenkemper T, Datta A, Kothare S, Riviello JJ Jr, Rotenberg A (2014). Electroencephalography in the pediatric emergency department: when is it most useful?. J Child Neurol.

[10] Kothare SV, Khurana DS, Valencia I, Melvin JJ, Legido A (2005). Use and value of ordering emergency electroencephalograms and videoelectroencephalographic monitoring after business hours in a children’s hospital: 1-year experience. J Child Neurol.

[11] Barcia Aguilar C, Sánchez Fernández I, Loddenkemper T (2020). Status epilepticus-work-up and management in children. Semin Neurol.

[12] Stephens CM, Mathieson SR, McNamara B (2023). Electroencephalography quality and application times in a pediatric emergency department setting: a feasibility study. Pediatr Neurol.

[13] Nozawa M, Terashima H, Tsuji S, Kubota M (2019). A simplified electroencephalogram monitoring system in the emergency room. Pediatr Emerg Care.

[14] Yamaguchi H, Nagase H, Nishiyama M (2019). Nonconvulsive seizure detection by reduced-lead electroencephalography in children with altered mental status in the emergency department. J Pediatr.

[15] Simma L, Bauder F, Schmitt-Mechelke T (2021). Feasibility and usefulness of rapid 2-channel-EEG-monitoring (point-of-care EEG) for acute CNS disorders in the paediatric emergency department: an observational study. Emerg Med J.

[16] Takase R, Sasaki R, Tsuji S, Uematsu S, Kubota M, Kobayashi T (2022). Benzodiazepine use for pediatric patients with suspected nonconvulsive status epilepticus with or without simplified electroencephalogram: a retrospective cohort study. Pediatr Emerg Care.

[17] Simma L, Kammerl A, Ramantani G (2025). Point-of-care EEG in the pediatric emergency department: a systematic review. Eur J Pediatr.

[18] Taran S, Ahmed W, Pinto R (2021). Educational initiatives for electroencephalography in the critical care setting: a systematic review and meta-analysis. Can J Anaesth.

[19] Legriel S, Jacq G, Lalloz A (2021). Teaching important basic EEG patterns of bedside electroencephalography to critical care staffs: a prospective multicenter study. Neurocrit Care.

[20] Chari G, Yadav K, Nishijima D, Omurtag A, Zehtabchi S (2019). Improving the ability of ED physicians to identify subclinical/electrographic seizures on EEG after a brief training module. Int J Emerg Med.

[21] Davey Z, Gupta PB, Li DR, Nayak RU, Govindarajan P (2022). Rapid response EEG: current state and future directions. Curr Neurol Neurosci Rep.

[22] Harris PA, Taylor R, Minor BL (2019). The REDCap consortium: building an international community of software platform partners. J Biomed Inform.

[23] Hirsch LJ, Fong MWK, Leitinger M (2021). American clinical neurophysiology society’s standardized critical care EEG terminology: 2021 version. J Clin Neurophysiol.

[24] Simma L, Romano F, Schmidt S, Ramantani G, Bölsterli BK (2023). Integrating neuromonitoring in pediatric emergency medicine: exploring two options for point-of-care electroencephalogram (pocEEG) via patient monitors-a technical note. J Pers Med.

[25] Lee HC, Jung CW (2018). Vital Recorder—a free research tool for automatic recording of high-resolution time-synchronised physiological data from multiple anaesthesia devices. Sci Rep.

[26] La Barge G (2007). Pre-and post-testing with more impact. J Ext.

[27] Eysenbach G (2004). Improving the quality of web surveys: the checklist for reporting results of internet e-surveys (CHERRIES). J Med Internet Res.

[28] Tamási B, Hothorn T (2021). tramME: mixed-effects transformation models using template model builder. R J.

[29] R Core Team (2021). R: A Language and Environment for Statistical Computing.

[30] O’Brien AJ, Brady RM (2016). Point-of-care ultrasound in paediatric emergency medicine. J Paediatr Child Health.

[31] Fernandez A, Asoodar M, van Kranen-Mastenbroek V, Majoie M, Balmer D (2025). What do you see? signature pedagogy in continuous electroencephalography teaching. J Clin Neurophysiol.

[32] Saloum D (2013). Critical skills: use them or lose them. Ann Emerg Med.

[33] Green SM, Ruben J (2009). Emergency department children are not as sick as adults: implications for critical care skills retention in an exclusively pediatric emergency medicine practice. J Emerg Med.

