# Supplementary material for: E-Learning for Pediatric Emergency Department Staff in Point-of-Care Electroencephalogram Interpretation: Prospective Cohort Study
Source: JMIR Med Educ. 2025 Aug 20;11:e69395. doi: 10.2196/69395 (PMC12370458; doi:10.2196/69395)

Supplementary figures

Figure S1: Histogram of the EEG total score at pretest (left panel), posttest (middle panel), and three month test (right panel). Median (dashed line), n=number of observations for each test

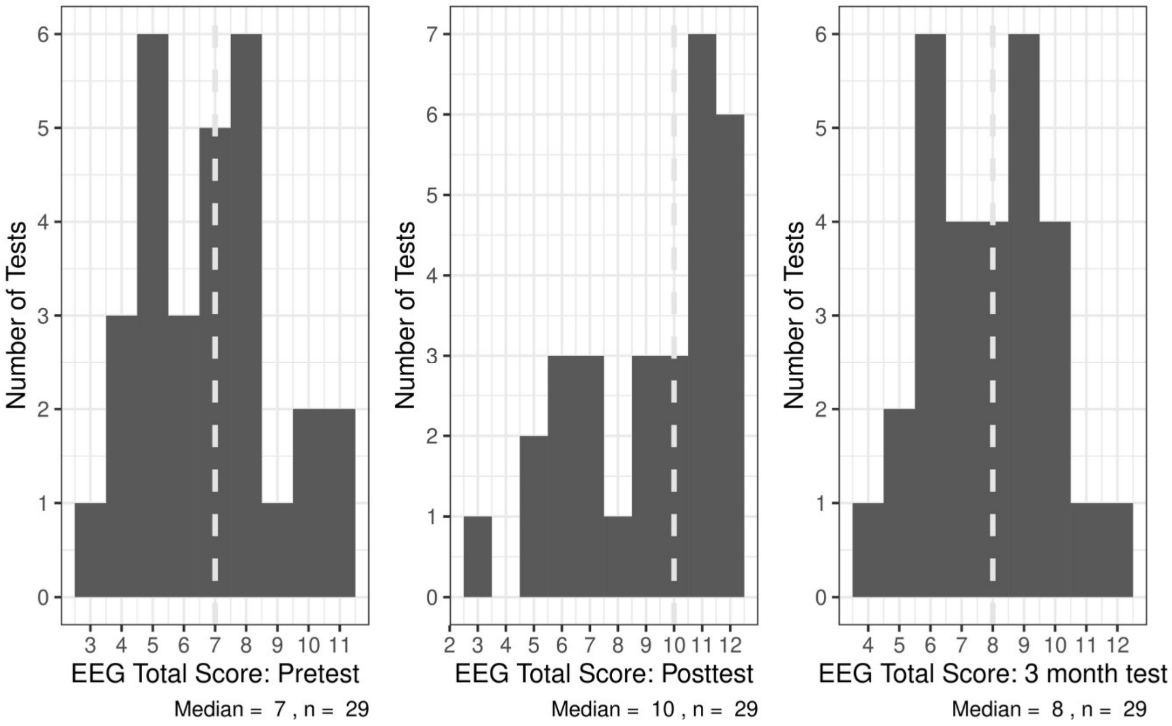

Figure S2: Confidence levels in applying pocEEG among participants of health care professionals (senior medical staff – SMS, junior medical staff – JMS and RN – registered nurses), measured on a 5-point Likert scale, responding to "I feel competent in applying a pocEEG".

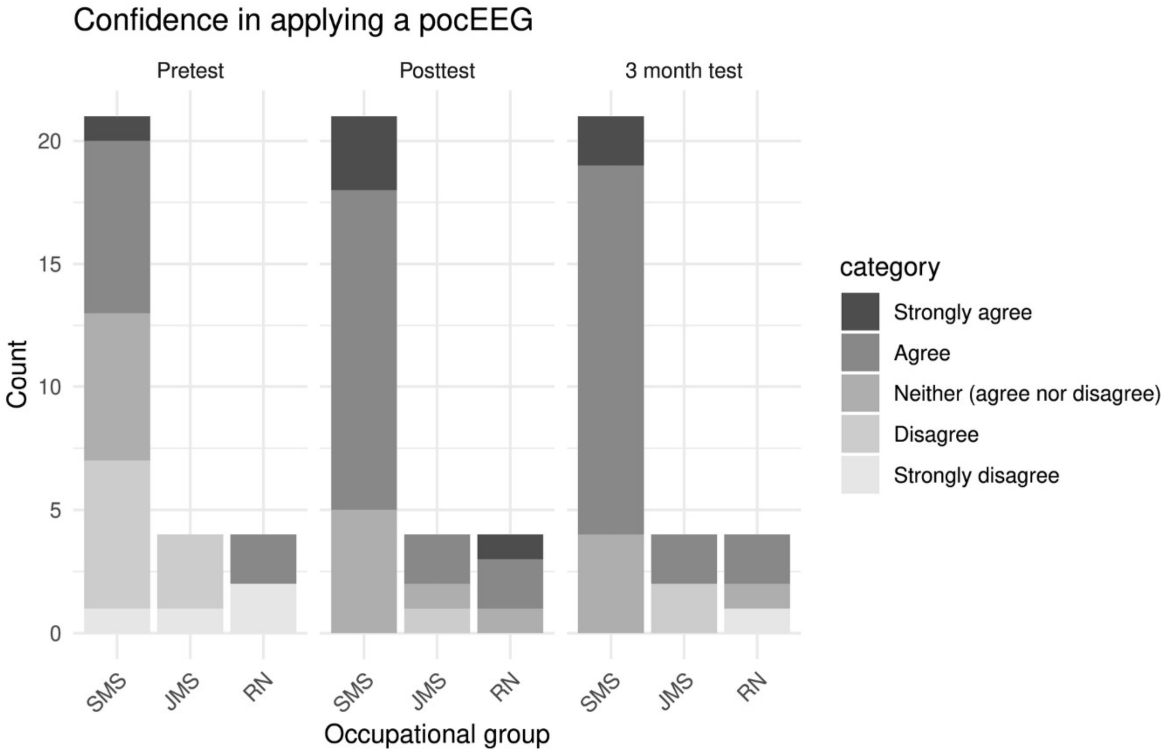

Figure S3: Confidence levels in detecting pocEEG among participants of health care professionals (senior medical staff – SMS, junior medical staff – JMS and RN – registered nurses), measured on a 5-point Likert scale, responding to “I feel competent in recognizing a pocEEG signal on the monitor”.

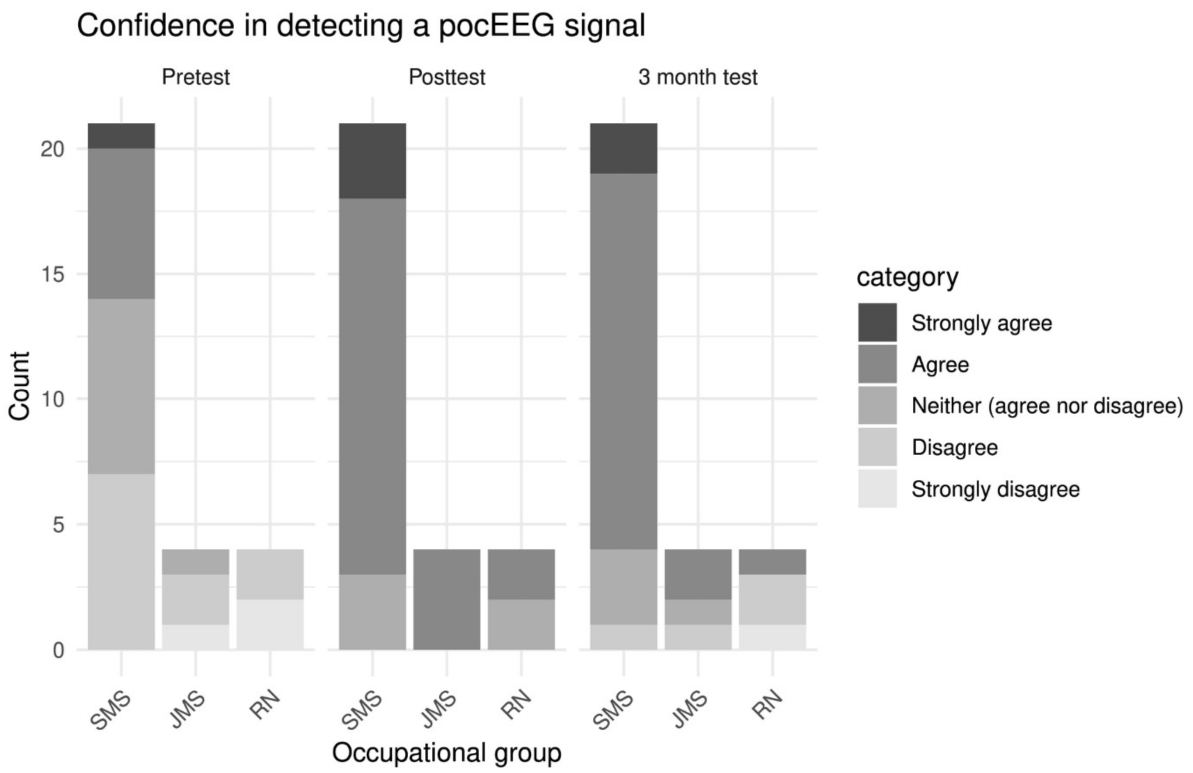

Figure S4: Confidence levels in interpreting a pocEEG signal among participants of health care professionals (senior medical staff – SMS, junior medical staff – JMS and RN – registered nurses), measured on a 5-point Likert scale, responding to "I feel confident interpreting a pocEEG".

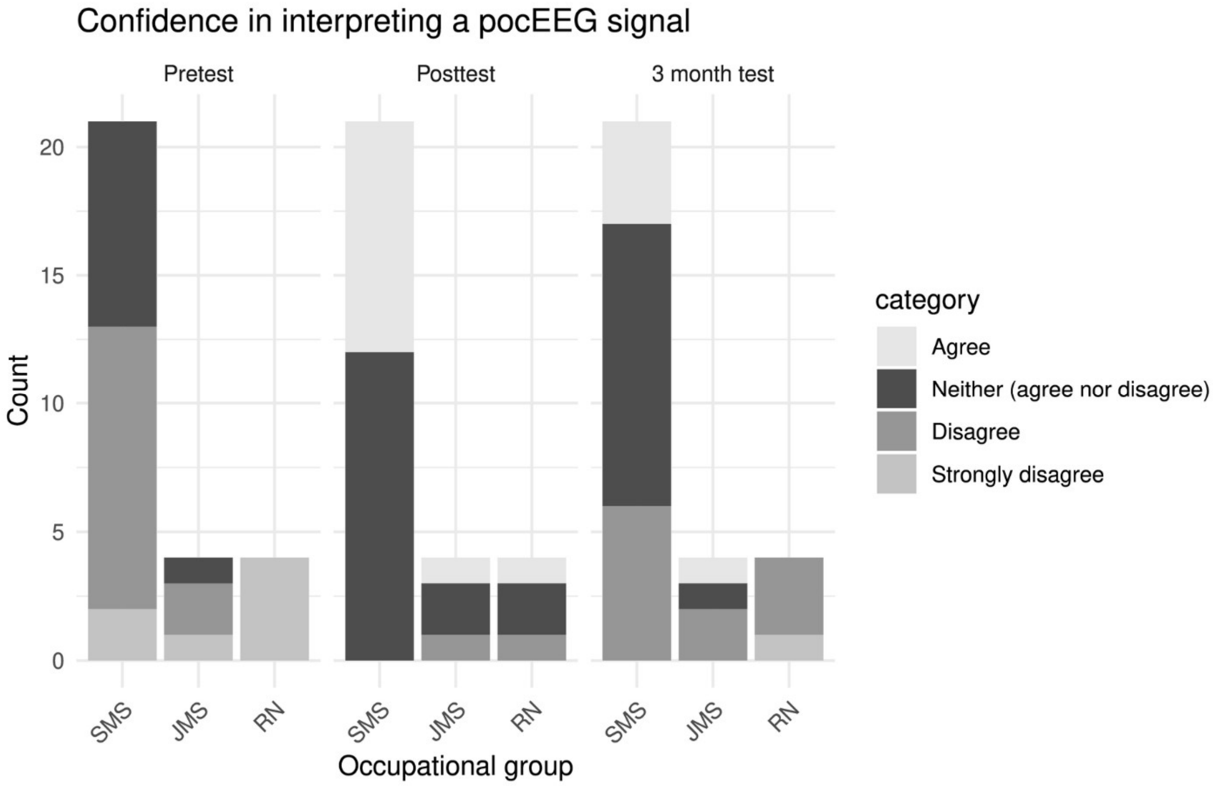

Figure S5: Confidence levels in detecting artifacts on pocEEG among participants of health care professionals (senior medical staff – SMS, junior medical staff – JMS and RN – registered nurses), measured on a 5-point Likert scale, responding to “I know which artifacts could appear on pocEEG”.

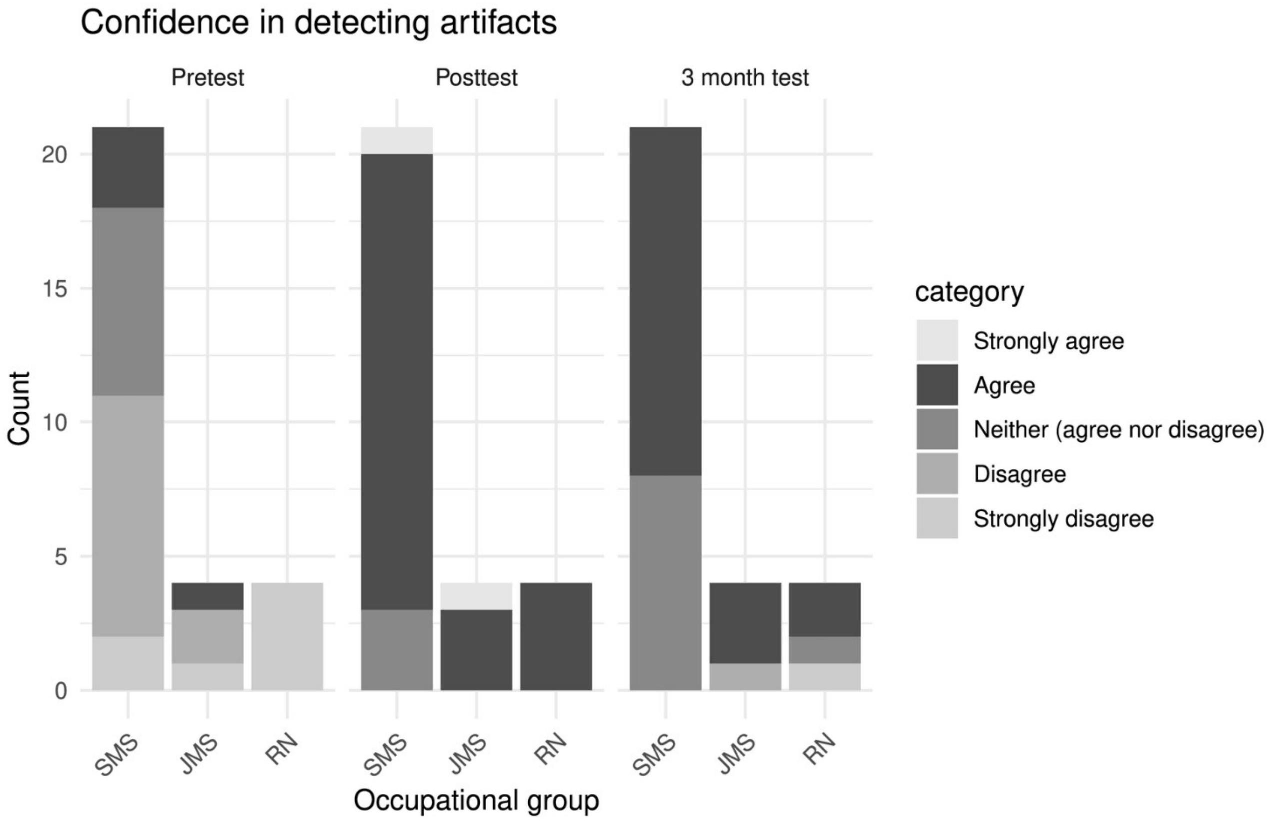

Figure S6: Confidence levels in addressing artifacts on pocEEG among participants of health care professionals (senior medical staff – SMS, junior medical staff – JMS and RN – registered nurses), measured on a 5-point Likert scale, responding to "I know how to address and fix artifacts".

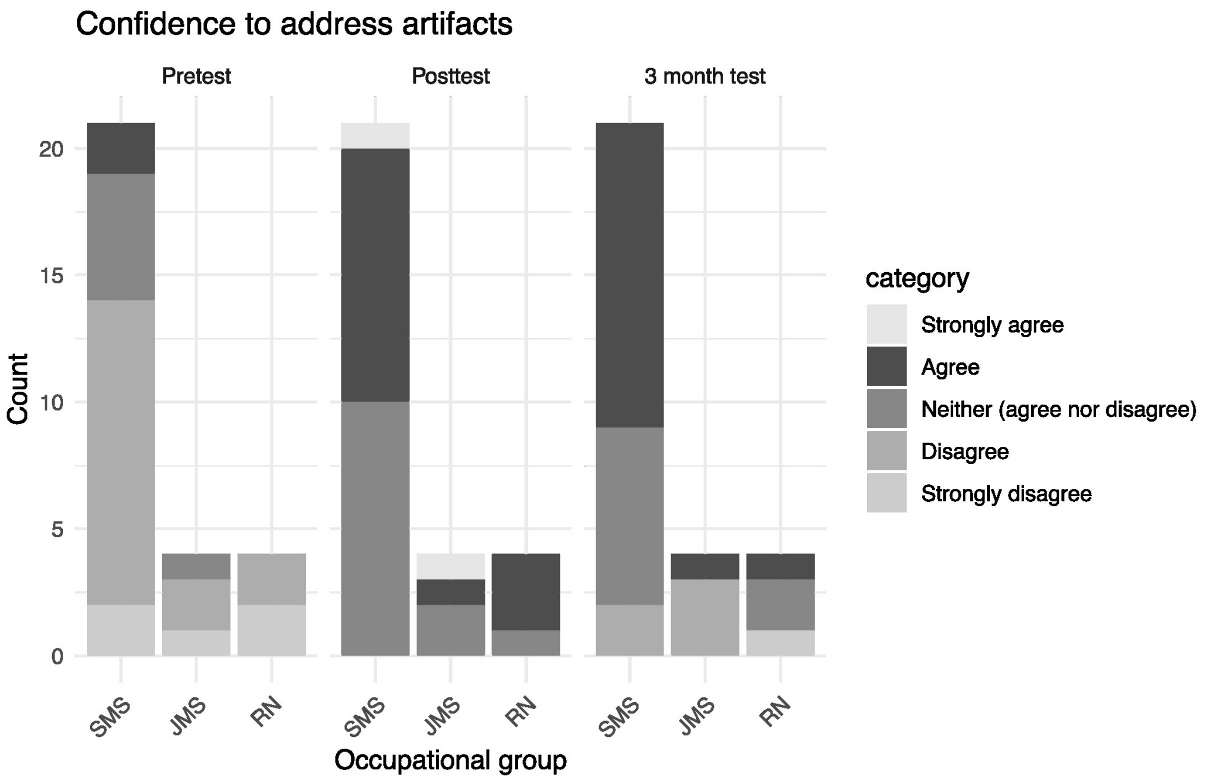

Supplement: Multimedia Appendix 1 [file mededu-v11-e69395-s001.pdf]
