# Supplementary material for: E-Learning for Pediatric Emergency Department Staff in Point-of-Care Electroencephalogram Interpretation: Prospective Cohort Study
Source: JMIR Med Educ. 2025 Aug 20;11:e69395. doi: 10.2196/69395 (PMC12370458; doi:10.2196/69395)
Supplement: Multimedia Appendix 2 [file mededu-v11-e69395-s002.zip › REDCap Codebook pocEEG learning module.pdf]

Data Dictionary Codebook

02-02-2023 10:58

#	Variable / Field Name	Field Label<br>*Field Note*	Field Attributes (Field Type, Validation, Choices, Calculations, etc.)
Instrument: **pocEEG Pretest** (poceeg_pretest) **Enabled as survey**			
1	[record_id]	Record ID	text
2	[vorwort]	Liebes Notfall-Team, Bitte füllt die multiple choice Fragen einfach nach eurem besten Wissen und Gewissen aus. Die Hypothese ist ja, dass wir als Notfallteam (noch) wenig darüber wissen. Der Test dient der Auswertung des Wissenszuwachses (und damit der Effektivität des Moduls). Im Anschluss an den Test erhaltet Ihr das Passwort für das Teaching Modul im Powerpoint Format.	descriptive
3	[consent]	Alle Personendaten sind für die Auswertung codiert, so dass sie nicht nach einzelnen Personen zugeordnet werden können. Die Teilnahme an der Studie ist freiwillig. Ihr könnt den Fragebogen jederzeit abbrechen. Deine Antworten werden nicht personenbezogen ausgewertet, die Emailadresse dient nur dem Zweck die Folgeumfragen zu versenden und dem richtigen Datensatz zuzuordnen (Vortest/Nachtest/3 Monate). Die Forschung folgt keinem kommerziellen Interesse. Die Daten werden streng vertraulich behandelt Ich erkläre hiermit, dass ich über den Inhalt und Zweck des Projekts informiert worden bin und bin einverstanden, am Projekt teilzunehmen.	radio, Required<br>1 Ja - ich stimme zu<br>2 Nein - ich lehne ab<br><br>Custom alignment: LV<br>Stop actions on 2
4	[sex_srvy]	Section Header:<br>Geschlecht	radio, Required<br>1 weiblich<br>2 männlich
5	[prf_srvy]	Berufsgruppe	radio, Required<br>1 Pflegefachperson Notfall<br>2 Ärztlicher Dienst Notfall (OAe, Fellow)<br>3 Ärztlicher Dienst Notfall (AAe)
6	[pilotparticipant]<br><br>Show the field ONLY if:<br>[prf_srvy] = '2'	Hast Du am Pilotquiz bzw. Einführungsfortbildung pocEEG (März 2021) teilgenommen?	radio, Required<br>1 ja<br>2 nein
7	[snrty]	Berufserfahrung in Jahren<br>- Pflege: ab Abschluss der Ausbildung<br>- Ärztlicher Dienst: ab Abschluss Facharzt oder wenn kein FA dann AAe wählen	radio, Required<br>1 0-5<br>2 5-10<br>3 10-15<br>4 15+<br>5 AAe - ohne FMH Titel
8	[pri_expr]	Section Header:<br>Hast du schon Erfahrung mit EEG und Interpretation	radio, Required<br>1 Nein<br>2 Nur davon gehört<br>3 Ja - aber nur aEEG auf der Neonatologie<br>4 Ja - schon ein paar Mal gemacht<br>5 Ja - und traue mir zu es zu interpretieren.
9	[cptc_appl]	Ich fühle mich kompetent ein pocEEG anzulegen bzw. die Anlage zu kontrollieren	radio (Matrix), Required<br>1 Stimme völlig zu<br>2 Stimme zu<br>3 Stimme weder zu noch nicht zu<br>4 Stimme nicht zu<br>5 Stimme überhaupt nicht zu

10	[cptc_sngl]	Ich fühle mich kompetent ein pocEEG Signal auf dem Monitor erkennen	radio (Matrix), Required<br>1 Stimme völlig zu<br>2 Stimme zu<br>3 Stimme weder zu noch nicht zu<br>4 Stimme nicht zu<br>5 Stimme überhaupt nicht zu
11	[cptc_intrp]	Ich fühle mich kompetent ein pocEEG zu interpretieren z.B. Status epilepticus	radio (Matrix), Required<br>1 Stimme völlig zu<br>2 Stimme zu<br>3 Stimme weder zu noch nicht zu<br>4 Stimme nicht zu<br>5 Stimme überhaupt nicht zu
12	[cptc_art]	Ich weiss welche Artefakte auftreten können	radio (Matrix), Required<br>1 Stimme völlig zu<br>2 Stimme zu<br>3 Stimme weder zu noch nicht zu<br>4 Stimme nicht zu<br>5 Stimme überhaupt nicht zu
13	[cptc_art_ex]	Ich weiss wie ich Artefakte beheben kann	radio (Matrix), Required<br>1 Stimme völlig zu<br>2 Stimme zu<br>3 Stimme weder zu noch nicht zu<br>4 Stimme nicht zu<br>5 Stimme überhaupt nicht zu
14	[utlty]	Aus jetziger Sicht: wie schätzt du die Nützlichkeit eines pocEEG auf der Notfallstation ein?	radio, Required<br>1 Äusserst hilfreich<br>2 Sehr hilfreich<br>3 Etwas hilfreich<br>4 Nur bedingt hilfreich<br>5 Überhaupt nicht hilfreich
15	[sy]	Section Header:<br>Was ist das internationale 10-20 System?	radio, Required<br>1 Es wurde zwischen 1910 und 1920 erfunden<br>2 Ableitungen mit 10 bis 20 Kanälen<br>888 Standardisierte Elektroden-Platzierung<br>4 Man benötigt nur 10 bis 20 Elektroden
16	[lat]	Section Header:<br>Gemäss 10-20 System für EEG-Ableitung, sind GERADE Zahlen (=2,4,6,...) welcher Seite des Gehirns zugeordnet?	radio, Required<br>1 Links<br>2 Frontal<br>888 Rechts<br>4 Temporal
17	[t_1020]	Was bedeutet Buchstabe T im 10-20 System?	radio, Required<br>888 Temporallappen<br>2 Thalamus<br>3 Tectum<br>4 Trigeminus
18	[f_1020]	Section Header:<br>Was bedeutet Buchstabe F im 10-20 System?	radio, Required<br>1 Facialis<br>888 Frontallappen<br>3 Falx<br>4 Fornix

19	[knwl_amp]	Welche Einheit beschreibt die Amplitude des EEG (die Höhe der Welle)?	radio, Required<br>1 Univolt<br>2 Milliampere (mA)<br>888 Mikrovolt (µV)<br>4 Hertz (Hz)<br>5 Kilowatt (kW)
20	[mc_instr]	Section Header:<br>Im nächsten Abschnitt kommen Fragen zur pocEEG Befunden.Grafik kann wie bei jedem Bild beschrieben bei Bedarf vergrössert werden, Videos ebenfalls via Symbol direkt im Videofeld. Es sind 12 Fragen.Es nur eine der fünf Antworten ist richtig. Hinweis: Achtet bei der Beantwortung auf stimmige Symmetrie UND Grundrhythmus des EEG (letztere sind bei Bedarf bei jeder Frage via Link einsehbar).	descriptive
21	[q1_poceeg]	Section Header: *pocEEG Befunde*<br>Frage 1 Falls Du das Bild grösser haben willst -> mit rechter Maustaste auf Bild klicken und "in neuem Tab öffnen" wählen	descriptive
22	[bsc_patterns1]	EEG Grundrhythmen nachschauen (auf Link klicken)	descriptive<br>Field Annotation: basic patterns for reference
23	[q1]	Frage 1: Die Ableitung zeigt	radio, Required<br>1 Symmetrie in Amplitude und Frequenz, Beta-Aktivität<br>2 Eingelagerte Artefakte von Muskelaktivität<br>3 Epileptische Entladungen auf beiden Ableitungen<br>4 Ausgeprägte Asymmetrie in Amplitude, Theta-Aktivität<br>888 Symmetrisch, Delta-Aktivität, EKG Artefakte
24	[guess_q1]	Bei der vorherigen Frage...	radio, Required<br>1 Ja - ich wusste die Antwort<br>2 Nein - ich habe geraten
25	[q2_poceeg]	Section Header:<br>Frage 2Falls Du das Bild grösser haben willst -> mit rechter Maustaste auf Bild klicken und "in neuem Tab öffnen" wählen	descriptive
26	[bsc_patterns2]	EEG Grundrhythmen nachschauen (auf Link klicken)	descriptive<br>Field Annotation: basic patterns for reference
27	[q2]	Frage 2: Die Ableitung zeigt	radio, Required<br>1 Ausgeprägte Asymmetrie in Amplitude und Frequenz, Elektrodenartefakte<br>888 Eingelagerte Artefakte von Augenbewegungen<br>3 Epileptische Anfallsmuster auf beiden Ableitungen<br>4 Asymmetrie in Amplitude, Theta-Aktivität<br>5 EKG Artefakte
28	[guess_q2]	Bei der vorherigen Frage...	radio, Required<br>1 Ja - ich wusste die Antwort<br>2 Nein - ich habe geraten
29	[q3_poceeg]	Section Header:<br>Frage 3Falls Du das Bild grösser haben willst -> mit rechter Maustaste auf Bild klicken und "in neuem Tab öffnen" wählen	descriptive
30	[bsc_patterns3]	EEG Grundrhythmen nachschauen (auf Link klicken)	descriptive<br>Field Annotation: basic patterns for reference

31	[q3]	Frage 3: Die Ableitung zeigt	radio, Required<br>1 Symmetrie in Amplitude und Frequenz, Beta-Aktivität Ableitung 1<br>2 Eingelagerte Artefakte von Augenbewegungen<br>3 Epileptische Anfallsmuster auf beiden Ableitungen<br>4 Asymmetrie in Amplitude, Delta-Aktivität Ableitung 2<br>888 Asymmetrie in Frequenz und Amplitude, Beta Aktivität-Ableitung 2
32	[guess_q3]	Bei der vorherigen Frage...	radio, Required<br>1 Ja - ich wusste die Antwort<br>2 Nein - ich habe geraten
33	[q4_poceeg]	Section Header:<br>Frage 4Falls Du das Bild grösser haben willst -> mit rechter Maustaste auf Bild klicken und "in neuem Tab öffnen" wählen	descriptive
34	[bsc_patterns4]	EEG Grundrhythmen nachschauen (auf Link klicken)	descriptive<br>Field Annotation: basic patterns for reference
35	[q4]	Frage 4: Die Ableitung auf dem Bild zeigt	radio, Required<br>1 Störartefakt von technischem Gerät<br>2 Symmetrisch in Frequenz und Amplitude, normale Delta-Aktivität<br>3 Leichte Asymmetrie in Amplitude, vorwiegend Beta-Aktivität<br>888 Deutliche Asymmetrie in Amplitude, epileptische Anfallsmuster<br>5 Normale kortikale Aktivität
36	[guess_q4]	Bei der vorherigen Frage...	radio, Required<br>1 Ja - ich wusste die Antwort<br>2 Nein - ich habe geraten
37	[q5_poceeg]	Section Header:<br>Frage 5	descriptive
38	[bsc_patterns5]	EEG Grundrhythmen nachschauen (auf Link klicken)	descriptive<br>Field Annotation: basic patterns for reference
39	[q5]	Frage 5: Die Ableitung zeigt	radio, Required<br>1 Symmetrie in Amplitude und Frequenz, Beta-Aktivität Ableitung 1<br>2 Eingelagerte Artefakte von Augenbewegungen<br>888 Anfallsmuster auf beiden Ableitungen<br>4 Leichte Asymmetrie in Amplitude, vorwiegend Theta-Aktivität<br>5 Ausgeprägte Asymmetrie in Frequenz, vorwiegend Beta-Aktivität
40	[guess_q5]	Bei der vorherigen Frage...	radio, Required<br>1 Ja - ich wusste die Antwort<br>2 Nein - ich habe geraten
41	[q6_poceeg]	Section Header:<br>Frage 6Falls Du das Bild (noch) grösser haben willst -> mit rechter Maustaste auf Bild klicken und "in neuem Tab öffnen" wählen	descriptive
42	[bsc_patterns6]	EEG Grundrhythmen nachschauen (auf Link klicken)	descriptive<br>Field Annotation: basic patterns for reference

43	[q6]	Frage 6: Die Ableitung zeigt	radio, Required<br>888 Symmetrisch, vorwiegende Delta-Aktivität, normales Schlaf-EEG.<br>2 Leichte Asymmetrie in Amplitude, Beta-Aktivität<br>4 Ausgeprägte Asymmetrie in Amplitude, Theta-Aktivität<br>5 Leichte Asymmetrie in Frequenz, Delta-Aktivität<br>3 Epileptische Anfallsmuster auf beiden Ableitungen
44	[guess_q6]	Bei der vorherigen Frage...	radio, Required<br>1 Ja - ich wusste die Antwort<br>2 Nein - ich habe geraten
45	[q7_poceeeg]	Section Header:<br>Frage 7:	descriptive
46	[q7]	Frage 7: Die Ableitung zeigt	radio, Required<br>1 Weitgehende Symmetrie in Amplitude und Frequenz, Theta-Delta Aktivität, vereinzelt epilepsietypische Potenziale<br>2 Ausgeprägte Asymmetrie in Amplitude und Frequenz, Beta-Aktivität<br>888 Epileptische Anfallsmuster in beiden Ableitungen<br>4 Ausgeprägte Asymmetrie in Amplitude, vorwiegend Alpha-Aktivität<br>5 leichte Asymmetrie in Frequenz, vorwiegend Beta-Aktivität
47	[bsc_patterns7]	EEG Grundrhythmen nachschauen (auf Link klicken)	descriptive<br>Field Annotation: basic patterns for reference
48	[guess_q7]	Bei der vorherigen Frage...	radio, Required<br>1 Ja - ich wusste die Antwort<br>2 Nein - ich habe geraten
49	[q8_poceeg]	Section Header:<br>Frage 8:	descriptive
50	[bsc_patterns8]	EEG Grundrhythmen nachschauen (auf Link klicken)	descriptive<br>Field Annotation: basic patterns for reference
51	[q8]	Frage 8: Die Ableitung zeigt	radio, Required<br>1 Symmetrie in Amplitude und Frequenz, Alpha-Aktivität, intermittierende epilepsietypische Potenziale<br>2 Leichte Asymmetrie in Amplitude, vorwiegend Beta-Aktivität<br>4 Ausgeprägte Asymmetrie in Amplitude, Theta-Aktivität<br>5 Leichte Asymmetrie in Frequenz, Delta-Aktivität<br>888 Hochamplitudige Spitzen auf beiden Ableitungen- Epileptisch oder Muskelartefakt - Korrelation mit Klinik nötig
52	[guess_q8]	Bei der vorherigen Frage...	radio, Required<br>1 Ja - ich wusste die Antwort<br>2 Nein - ich habe geraten
53	[q9_poceeg]	Section Header:<br>Frage 9:	descriptive
54	[bsc_patterns9]	EEG Grundrhythmen nachschauen (auf Link klicken)	descriptive<br>Field Annotation: basic patterns for reference

55	[q9]	Frage 9: Die Ableitung zeigt	radio, Required<br>888 Symmetrie in Amplitude und Frequenz, vorwiegend Delta-Theta Mischaktivität, keine Anfallsmuster<br>2 Ausgeprägte Asymmetrie in Amplitude, Beta-Aktivität, St. n. Benzodiazepin-Gabe<br>4 Ausgeprägte Asymmetrie in Frequenz, Alpha-Aktivität<br>5 leichte Asymmetrie in Frequenz, Subdelta-Aktivität, epilepsietypische Potenziale<br>3 Epileptische Anfallsmuster auf Ableitung 1
56	[guess_q9]	Bei der vorherigen Frage...	radio, Required<br>1 Ja - ich wusste die Antwort<br>2 Nein - ich habe geraten
57	[q10_poceeg]	Section Header:<br>Frage 10: Falls Du das Bild grösser haben willst -> mit rechter Maustaste auf Bild klicken und "in neuem Tab öffnen" wählen	descriptive
58	[bsc_patterns10]	EEG Grundrhythmen nachschauen (auf Link klicken)	descriptive<br>Field Annotation: basic patterns for reference
59	[q10]	Frage 10: Die Ableitung zeigt	radio, Required<br>1 Symmetrie in Amplitude und Frequenz, Theta-Aktivität, epilepsietypische Potenziale<br>2 Leichte Asymmetrie in Amplitude, Beta-Aktivität<br>4 Ausgeprägte Asymmetrie in Amplitude, Alpha-Aktivität<br>888 Symmetrie in Amplitude und Frequenz, Delta-Theta Aktivität, Kind schläft wahrscheinlich (klinisch schauen)<br>3 Ausgeprägte Asymmetrie, epileptische Anfallsmuster auf Ableitung 2
60	[guess_q10]	Bei der vorherigen Frage...	radio, Required<br>1 Ja - ich wusste die Antwort<br>2 Nein - ich habe geraten
61	[q11_poceeg]	Section Header:<br>Frage 11:<br><br>Falls Du das Bild grösser haben willst -> mit rechter Maustaste auf Bild klicken und "in neuem Tab öffnen" wählen	descriptive
62	[bsc_patterns11]	EEG Grundrhythmen nachschauen (auf Link klicken)	descriptive<br>Field Annotation: basic patterns for reference
63	[q11]	Frage 11: Die Ableitung zeigt	radio, Required<br>1 Symmetrie in Amplitude und Frequenz, Theta-Aktivität intermittierende epilepsietypische Potenziale<br>2 Leichte Asymmetrie in Amplitude und Frequenz, Delta-Aktivität, Vigilanz beurteilen<br>888 Ausgeprägte Asymmetrie bezüglich Amplitude, Symmetrie hinsichtlich Frequenz, Zur Beurteilung ob echt: Elektroden testen.<br>5 Leichte Asymmetrie in Frequenz, Delta-Aktivität<br>3 Symmetrisch in Frequenz und Amplitude, mit EKG Artefakten, Alpha-Aktivität
64	[guess_q11]	Bei der vorherigen Frage...	radio, Required<br>1 Ja - ich wusste die Antwort<br>2 Nein - ich habe geraten
65	[q12_poceeg]	Section Header:<br>Frage 12:	descriptive
66	[bsc_patterns12]	EEG Grundrhythmen nachschauen (auf Link klicken)	descriptive<br>Field Annotation: basic patterns for reference

67	[q12]	Frage 12: Die Ableitung zeigt	radio, Required<br>1 Symmetrie in Amplitude und Frequenz, vereinzelte epilepsietypische Potenziale<br>2 Ausgeprägte Asymmetrie in Amplitude, symmetrische Frequenz, vorwiegend Beta-Aktivität<br>888 Symmetrisch in Amplitude und Frequenz, vorwiegend Delta-Theta Aktivität<br>5 leichte Asymmetrie in Frequenz, Beta Aktivität<br>3 Symmetrie Amplitude und Frequenz, Bewegungsartefakte
68	[guess_q12]	Bei der vorherigen Frage...	radio, Required<br>1 Ja - ich wusste die Antwort<br>2 Nein - ich habe geraten
69	[bsc_knwl]	Section Header: *Ende Test*<br>Correct basic knowledge pretest (out of 5)	calc<br>Calculation: sum([basic_knw_1],[basic_knw_2],[basic_knw_3],[basic_knw_4],[basic_knw_5])<br>Field Annotation: @HIDDEN
70	[total_guessrte]	Total guessrate (nein ich habe geraten) out of 12	calc<br>Calculation: sum([gsrte1],[gsrte2],[gsrte3],[gsrte4],[gsrte5],[gsrte6],[gsrte7],[gsrte8],[gsrte9],[gsrte10],[gsrte11],[gsrte12])<br>Field Annotation: @HIDDEN
71	[total_ngss_corr]	Total no guess&correct	calc<br>Calculation: sum([nogss_corr_1],[nogss_corr_2],[nogss_corr_3],[nogss_corr_4],[nogss_corr_5],[nogss_corr_5],[nogss_corr_6],[nogss_corr_7],[nogss_corr_8],[nogss_corr_9],[nogss_corr_10],[nogss_corr_11],[nogss_corr_12])<br>Field Annotation: @HIDDEN
72	[eeg_total_correct]	Total correct out of 12	calc<br>Calculation: sum([q1_corr],[q2_corr],[q3_corr],[q4_corr],[q5_corr],[q6_corr],[q7_corr],[q8_corr],[q9_corr],[q10_corr],[q11_corr],[q12_corr])<br>Field Annotation: @HIDDEN
73	[eeg_artif_corr]	Korrekte Artefakt pocEEG out of 4	calc<br>Calculation: sum([q1_corr],[q2_corr],[q8_corr],[q11_corr])<br>Field Annotation: @HIDDEN Artefakte q1-2-8-11
74	[eeg_path_corr]	Korrekte pathologische pocEEG out of 4	calc<br>Calculation: sum([q3_corr],[q4_corr],[q5_corr],[q7_corr])<br>Field Annotation: @HIDDEN
75	[eeg_norm_corr]	Korrekte normale pocEEG out of 4	calc<br>Calculation: sum([q6_corr],[q9_corr],[q10_corr],[q12_corr])<br>Field Annotation: @HIDDEN
76	[gsrte1]	Guess rate out of 1	calc<br>Calculation: if ([guess_q1] = 2,1,0)<br>Field Annotation: @HIDDEN
77	[gsrte2]	Guess rate 2	calc<br>Calculation: if ([guess_q2] = 2,1,0)<br>Field Annotation: @HIDDEN
78	[gsrte3]	Guess rate 3	calc<br>Calculation: if ([guess_q3] = 2,1,0)<br>Field Annotation: @HIDDEN
79	[gsrte4]	Guess rate 4	calc<br>Calculation: if ([guess_q4] = 2,1,0)<br>Field Annotation: @HIDDEN
80	[gsrte5]	Guess rate 5	calc<br>Calculation: if ([guess_q5] = 2,1,0)<br>Field Annotation: @HIDDEN
81	[gsrte6]	Guess rate 6	calc<br>Calculation: if ([guess_q6] = 2,1,0)<br>Field Annotation: @HIDDEN
82	[gsrte7]	Guess rate 7	calc<br>Calculation: if ([guess_q7] = 2,1,0)<br>Field Annotation: @HIDDEN

83	[gsrte8]	Guess rate 8	calc<br>Calculation: if ([guess_q8] = 2,1,0)<br>Field Annotation: @HIDDEN
84	[gsrte9]	Guess rate 9	calc<br>Calculation: if ([guess_q9] = 2,1,0)<br>Field Annotation: @HIDDEN
85	[gsrte10]	Guess rate 10	calc<br>Calculation: if ([guess_q10] = 2,1,0)<br>Field Annotation: @HIDDEN
86	[gsrte11]	Guess rate 11	calc<br>Calculation: if ([guess_q11] = 2,1,0)<br>Field Annotation: @HIDDEN
87	[gsrte12]	Guess rate 12	calc<br>Calculation: if ([guess_q12] = 2,1,0)<br>Field Annotation: @HIDDEN
88	[q1_corr]	q1 correct answer	calc<br>Calculation: if([q1] = 888, 1, 0)<br>Field Annotation: @HIDDEN
89	[q2_corr]	q2 correct answer	calc<br>Calculation: if([q2] = 888, 1, 0)<br>Field Annotation: @HIDDEN
90	[q3_corr]	q3 correct answer	calc<br>Calculation: if([q3] = 888, 1, 0)<br>Field Annotation: @HIDDEN
91	[q4_corr]	q4 correct answer	calc<br>Calculation: if([q4] = 888, 1, 0)<br>Field Annotation: @HIDDEN
92	[q5_corr]	q5 correct answer	calc<br>Calculation: if([q5] = 888, 1, 0)<br>Field Annotation: @HIDDEN
93	[q6_corr]	q6 correct answer	calc<br>Calculation: if([q6] = 888, 1, 0)<br>Field Annotation: @HIDDEN
94	[q7_corr]	q7 correct answer	calc<br>Calculation: if([q7] = 888, 1, 0)<br>Field Annotation: @HIDDEN
95	[q8_corr]	q8 correct answer	calc<br>Calculation: if([q8] = 888, 1, 0)<br>Field Annotation: @HIDDEN
96	[q9_corr]	q9 correct answer	calc<br>Calculation: if([q9] = 888, 1, 0)<br>Field Annotation: @HIDDEN
97	[q10_corr]	q10 correct answer	calc<br>Calculation: if([q10] = 888, 1, 0)<br>Field Annotation: @HIDDEN
98	[q11_corr]	q11 correct answer	calc<br>Calculation: if([q11] = 888, 1, 0)<br>Field Annotation: @HIDDEN
99	[q12_corr]	q12 correct answer	calc<br>Calculation: if([q12] = 888, 1, 0)<br>Field Annotation: @HIDDEN
100	[basic_knw_1]	q sy correct answer	calc<br>Calculation: if([sy] = 888, 1, 0)<br>Field Annotation: @HIDDEN
101	[basic_knw_2]	q lat correct answer	calc<br>Calculation: if([lat] = 888, 1, 0)<br>Field Annotation: @HIDDEN
102	[basic_knw_3]	q t_1020 correct answer	calc<br>Calculation: if([t_1020] = 888, 1, 0)<br>Field Annotation: @HIDDEN
103	[basic_knw_4]	q f_1020 correct answer	calc<br>Calculation: if([f_1020] = 888, 1, 0)<br>Field Annotation: @HIDDEN
104	[basic_knw_5]	q t_1020 correct answer	calc<br>Calculation: if([knwl_amp] = 888, 1, 0)<br>Field Annotation: @HIDDEN

105	[nogss_corr_1]	no_guess_corr_q1	calc<br>Calculation: if([q1] = 888 AND [guess_q1] = 1, 1, 0)<br>Field Annotation: @HIDDEN
106	[nogss_corr_2]	no_guess_corr_q2	calc<br>Calculation: if([q2] = 888 AND [guess_q2] = 1, 1, 0)<br>Field Annotation: @HIDDEN
107	[nogss_corr_3]	no_guess_corr_q3	calc<br>Calculation: if([q3] = 888 AND [guess_q3] = 1, 1, 0)<br>Field Annotation: @HIDDEN
108	[nogss_corr_4]	no_guess_corr_q4	calc<br>Calculation: if([q4] = 888 AND [guess_q4] = 1, 1, 0)<br>Field Annotation: @HIDDEN
109	[nogss_corr_5]	no_guess_corr_q5	calc<br>Calculation: if([q5] = 888 AND [guess_q5] = 1, 1, 0)<br>Field Annotation: @HIDDEN
110	[nogss_corr_6]	no_guess_corr_q6	calc<br>Calculation: if([q5] = 888 AND [guess_q5] = 1, 1, 0)<br>Field Annotation: @HIDDEN
111	[nogss_corr_7]	no_guess_corr_q7	calc<br>Calculation: if([q7] = 888 AND [guess_q7] = 1, 1, 0)<br>Field Annotation: @HIDDEN
112	[nogss_corr_8]	no_guess_corr_q8	calc<br>Calculation: if([q8] = 888 AND [guess_q8] = 1, 1, 0)<br>Field Annotation: @HIDDEN
113	[nogss_corr_9]	no_guess_corr_q9	calc<br>Calculation: if([q9] = 888 AND [guess_q9] = 1, 1, 0)<br>Field Annotation: @HIDDEN
114	[nogss_corr_10]	no_guess_corr_q10	calc<br>Calculation: if([q10] = 888 AND [guess_q10] = 1, 1, 0)<br>Field Annotation: @HIDDEN
115	[nogss_corr_11]	no_guess_corr_q11	calc<br>Calculation: if([q11] = 888 AND [guess_q11] = 1, 1, 0)<br>Field Annotation: @HIDDEN
116	[nogss_corr_12]	no_guess_corr_q12	calc<br>Calculation: if([q12] = 888 AND [guess_q12] = 1, 1, 0)<br>Field Annotation: @HIDDEN
117	[poceeg_pretest_complete]	Section Header: *Form Status*<br>Complete?	dropdown<br>0 Incomplete<br>1 Unverified<br>2 Complete
**Instrument: pocEEG Posttest (poceeg_posttest) Enabled as survey**			
118	[vorwort_v2]	Liebes Notfall-Team, Willkommen zum pocEEG - Lernmodul Post-Test Bitte füllt die multiple choice Fragen einfach nach eurem besten Wissen und Gewissen aus....	descriptive
119	[y_n_module]	Section Header:<br>Ich habe das Lernmodul gemacht	radio, Required<br>1 ja<br>2 nein
120	[module_no_4444]	Bitte gib die 4- stellige Zahl aus dem Lernmodul zur Bestätigung ein.	text (integer, Min: 1000, Max: 9999), Required
121	[cptc_appl_v2]	Section Header:<br>Ich fühle mich kompetent ein pocEEG anzulegen bzw. die Anlage zu kontrollieren	radio (Matrix), Required<br>1 Stimme völlig zu<br>2 Stimme zu<br>3 Stimme weder zu noch nicht zu<br>4 Stimme nicht zu<br>5 Stimme überhaupt nicht zu
122	[cptc_sngl_v2]	Ich fühle mich kompetent ein pocEEG Signal auf dem Monitor erkennen	radio (Matrix), Required<br>1 Stimme völlig zu<br>2 Stimme zu<br>3 Stimme weder zu noch nicht zu<br>4 Stimme nicht zu<br>5 Stimme überhaupt nicht zu

123	[cptc_intrp_v2]	Ich fühle mich kompetent ein pocEEG zu interpretieren z.B. Status epilepticus	radio (Matrix), Required<br>1 Stimme völlig zu<br>2 Stimme zu<br>3 Stimme weder zu noch nicht zu<br>4 Stimme nicht zu<br>5 Stimme überhaupt nicht zu
124	[cptc_art_v2]	Ich weiss welche Artefakte auftreten können	radio (Matrix), Required<br>1 Stimme völlig zu<br>2 Stimme zu<br>3 Stimme weder zu noch nicht zu<br>4 Stimme nicht zu<br>5 Stimme überhaupt nicht zu
125	[cptc_art_ex_v2]	Ich weiss wie ich Artefakte beheben kann	radio (Matrix), Required<br>1 Stimme völlig zu<br>2 Stimme zu<br>3 Stimme weder zu noch nicht zu<br>4 Stimme nicht zu<br>5 Stimme überhaupt nicht zu
126	[utlty_v2]	Aus jetziger Sicht: wie schätzt du die Nützlichkeit eines pocEEG auf der Notfallstation ein?	radio, Required<br>1 Äusserst hilfreich<br>2 Sehr hilfreich<br>3 Etwas hilfreich<br>4 Nur bedingt hilfreich<br>5 Überhaupt nicht hilfreich
127	[sy_v2]	Section Header:<br>Was ist das internationale 10-20 System?	radio, Required<br>1 Es wurde zwischen 1910 und 1920 erfunden<br>2 Ableitungen mit 10 bis 20 Kanälen<br>888 Standardisierte Elektroden-Platzierung<br>4 Man benötigt nur 10 bis 20 Elektroden
128	[lat_v2]	Section Header:<br>Gemäss 10-20 System für EEG-Ableitung, sind GERADE Zahlen (=2,4,6,...) welcher Seite des Gehirns zugeordnet?	radio, Required<br>1 Links<br>2 Frontal<br>888 Rechts<br>4 Temporal
129	[t_1020_v2]	Was bedeutet Buchstabe T im 10-20 System?	radio, Required<br>888 Temporallappen<br>2 Thalamus<br>3 Tectum<br>4 Trigeminus
130	[f_1020_v2]	Section Header:<br>Was bedeutet Buchstabe F im 10-20 System?	radio, Required<br>1 Facialis<br>888 Frontallappen<br>3 Falx<br>4 Fornix
131	[knwl_amp_v2]	Welche Einheit beschreibt die Amplitude des EEG (die Höhe der Welle)?	radio, Required<br>1 Univolt<br>2 Milliampere (mA)<br>888 Mikrovolt (μV)<br>4 Hertz (Hz)<br>5 Kilowatt (kW)

132	[mc_instr_2]	Section Header:<br>Im nächsten Abschnitt kommen Fragen zur pocEEG Befunden.Grafik kann wie bei jedem Bild beschrieben bei Bedarf vergrössert werden, Videos ebenfalls via Symbol direkt im Videofeld. Es sind 12 Fragen.Es nur eine der fünf Antworten ist richtig. Hinweis: Achtet bei der Beantwortung auf stimmige Symmetrie UND Grundrhythmus des EEG (letztere sind bei Bedarf bei jeder Frage via Link einsehbar).	descriptive
133	[q3_poceeg_v2]	Section Header: *pocEEG Befunde*<br>Frage 1Falls Du das Bild grösser haben willst -> mit rechter Maustaste auf Bild klicken und "in neuem Tab öffnen" wählen	descriptive
134	[bsc_patterns3_v2]	EEG Grundrhythmen nachschauen (auf Link klicken)	descriptive<br>Field Annotation: basic patterns for reference
135	[q3_v2]	Frage 1: Die Ableitung zeigt	radio, Required<br>1 | Symmetrie in Amplitude und Frequenz, Beta-Aktivität Ableitung 1<br>2 | Eingelagerte Artefakte von Augenbewegungen<br>3 | Epileptische Anfallsmuster auf beiden Ableitungen<br>4 | Asymmetrie in Amplitude, Delta-Aktivität Ableitung 2<br>888 | Asymmetrie in Frequenz, Beta Aktivität-Ableitung 2
136	[guess_q3_v2]	Bei der vorherigen Frage...	radio, Required<br>1 | Ja - ich wusste die Antwort<br>2 | Nein - ich habe geraten
137	[q2_poceeg_v2]	Section Header:<br>Frage 2Falls Du das Bild grösser haben willst -> mit rechter Maustaste auf Bild klicken und "in neuem Tab öffnen" wählen	descriptive
138	[bsc_patterns2_v2]	EEG Grundrhythmen nachschauen (auf Link klicken)	descriptive<br>Field Annotation: basic patterns for reference
139	[q2_v2]	Frage 2: Die Ableitung zeigt	radio, Required<br>1 | Ausgeprägte Asymmetrie in Amplitude und Frequenz, Elektrodenartefakte<br>888 | Eingelagerte Artefakte von Augenbewegungen<br>3 | Epileptische Anfallsmuster auf beiden Ableitungen<br>4 | Asymmetrie in Amplitude, Theta-Aktivität<br>5 | EKG Artefakte
140	[guess_q2_v2]	Bei der vorherigen Frage...	radio, Required<br>1 | Ja - ich wusste die Antwort<br>2 | Nein - ich habe geraten
141	[q4_poceeg_v2]	Section Header:<br>Frage 3Falls Du das Bild grösser haben willst -> mit rechter Maustaste auf Bild klicken und "in neuem Tab öffnen" wählen	descriptive
142	[bsc_patterns4_v2]	EEG Grundrhythmen nachschauen (auf Link klicken)	descriptive<br>Field Annotation: basic patterns for reference
143	[q4_v2]	Frage 3: Die Ableitung auf dem Bild zeigt	radio, Required<br>1 | Störartefakt von technischem Gerät<br>2 | Symmetrisch in Frequenz und Amplitude, normale Delta-Aktivität<br>3 | Leichte Asymmetrie in Amplitude, vorwiegend Beta-Aktivität<br>888 | Deutliche Asymmetrie in Amplitude, epileptische Anfallsmuster<br>5 | Normale kortikale Aktivität
144	[guess_q4_v2]	Bei der vorherigen Frage...	radio, Required<br>1 | Ja - ich wusste die Antwort<br>2 | Nein - ich habe geraten

145	[q6_poceeg_v2]	Section Header:<br>Frage 4Falls Du das Bild (noch) grösser haben willst -> mit rechter Maustaste auf Bild klicken und "in neuem Tab öffnen" wählen	descriptive
146	[bsc_patterns6_v2]	EEG Grundrhythmen nachschauen (auf Link klicken)	descriptive<br>Field Annotation: basic patterns for reference
147	[q6_v2]	Frage 4: Die Ableitung zeigt	radio, Required<br>888 Symmetrisch, vorwiegende Delta-Aktivität, normales Schlaf-EEG.<br>2 Leichte Asymmetrie in Amplitude, Beta-Aktivität<br>4 Ausgeprägte Asymmetrie in Amplitude, Theta-Aktivität<br>5 Leichte Asymmetrie in Frequenz, Delta-Aktivität<br>3 Epileptische Anfallsmuster auf beiden Ableitungen
148	[guess_q6_v2]	Bei der vorherigen Frage...	radio, Required<br>1 Ja - ich wusste die Antwort<br>2 Nein - ich habe geraten
149	[q5_poceeg_v2]	Section Header:<br>Frage 5	descriptive
150	[bsc_patterns5_v2]	EEG Grundrhythmen nachschauen (auf Link klicken)	descriptive<br>Field Annotation: basic patterns for reference
151	[q5_v2]	Frage 5: Die Ableitung zeigt	radio, Required<br>1 Symmetrie in Amplitude und Frequenz, Beta-Aktivität Ableitung 1<br>2 Eingelagerte Artefakte von Augenbewegungen<br>888 Anfallsmuster auf beiden Ableitungen<br>4 Leichte Asymmetrie in Amplitude, vorwiegend Theta-Aktivität<br>5 Ausgeprägte Asymmetrie in Frequenz, vorwiegend Beta-Aktivität
152	[guess_q5_v2]	Bei der vorherigen Frage...	radio, Required<br>1 Ja - ich wusste die Antwort<br>2 Nein - ich habe geraten
153	[q7_poceeeg_v2]	Section Header:<br>Frage 6:	descriptive
154	[bsc_patterns7_v2]	EEG Grundrhythmen nachschauen (auf Link klicken)	descriptive<br>Field Annotation: basic patterns for reference
155	[q7_v2]	Frage 6: Die Ableitung zeigt	radio, Required<br>1 Weitgehende Symmetrie in Amplitude und Frequenz, Theta-Delta Aktivität, vereinzelt epilepsietypische Potenziale<br>2 Ausgeprägte Asymmetrie in Amplitude und Frequenz, Beta-Aktivität<br>888 Epileptische Anfallsmuster in beiden Ableitungen<br>4 Ausgeprägte Asymmetrie in Amplitude, vorwiegend Alpha-Aktivität<br>5 leichte Asymmetrie in Frequenz, vorwiegend Beta-Aktivität
156	[guess_q7_v2]	Bei der vorherigen Frage...	radio, Required<br>1 Ja - ich wusste die Antwort<br>2 Nein - ich habe geraten
157	[q8_poceeg_v2]	Section Header:<br>Frage 7:	descriptive
158	[bsc_patterns8_v2]	EEG Grundrhythmen nachschauen (auf Link klicken)	descriptive<br>Field Annotation: basic patterns for reference

159	[q8_v2]	Frage 7: Die Ableitung zeigt	radio, Required<br>1 Symmetrie in Amplitude und Frequenz, Alpha-Aktivität, intermittierende epilepsietypische Potenziale<br>2 Leichte Asymmetrie in Amplitude, vorwiegend Beta-Aktivität<br>4 Ausgeprägte Asymmetrie in Amplitude, Theta-Aktivität<br>5 Leichte Asymmetrie in Frequenz, Delta-Aktivität<br>888 Hochamplitudige Spitzen auf beiden Ableitungen- Epileptisch oder Muskelartefakt - Korrelation mit Klinik nötig
160	[guess_q8_v2]	Bei der vorherigen Frage...	radio, Required<br>1 Ja - ich wusste die Antwort<br>2 Nein - ich habe geraten
161	[q1_poceeg_v2]	Section Header:<br>Frage 8Falls Du das Bild grösser haben willst -> mit rechter Maustaste auf Bild klicken und "in neuem Tab öffnen" wählen	descriptive
162	[bsc_patterns1_v2]	EEG Grundrhythmen nachschauen (auf Link klicken)	descriptive<br>Field Annotation: basic patterns for reference
163	[q1_v2]	Frage 8: Die Ableitung zeigt	radio, Required<br>1 Symmetrie in Amplitude und Frequenz, Beta-Aktivität<br>2 Eingelagerte Artefakte von Muskelaktivität<br>3 Epileptische Entladungen auf beiden Ableitungen<br>4 Ausgeprägte Asymmetrie in Amplitude, Theta-Aktivität<br>888 Symmetrisch, Delta-Aktivität, EKG Artefakte
164	[guess_q1_v2]	Bei der vorherigen Frage...	radio, Required<br>1 Ja - ich wusste die Antwort<br>2 Nein - ich habe geraten
165	[q9_poceeg_v2]	Section Header:<br>Frage 9:	descriptive
166	[bsc_patterns9_v2]	EEG Grundrhythmen nachschauen (auf Link klicken)	descriptive<br>Field Annotation: basic patterns for reference
167	[q9_v2]	Frage 9: Die Ableitung zeigt	radio, Required<br>888 Symmetrie in Amplitude und Frequenz, vorwiegend Delta-Theta Mischaktivität, keine Anfallsmuster<br>2 Ausgeprägte Asymmetrie in Amplitude, Beta-Aktivität, St. n. Benzodiazepin-Gabe<br>4 Ausgeprägte Asymmetrie in Frequenz, Alpha-Aktivität<br>5 leichte Asymmetrie in Frequenz, Subdelta-Aktivität, epilepsietypische Potenziale<br>3 Epileptische Anfallsmuster auf Ableitung 1
168	[guess_q9_v2]	Bei der vorherigen Frage...	radio, Required<br>1 Ja - ich wusste die Antwort<br>2 Nein - ich habe geraten
169	[q11_poceeg_v2]	Section Header:<br>Frage 10:<br><br>Falls Du das Bild grösser haben willst -> mit rechter Maustaste auf Bild klicken und "in neuem Tab öffnen" wählen	descriptive
170	[bsc_patterns11_v2]	EEG Grundrhythmen nachschauen (auf Link klicken)	descriptive<br>Field Annotation: basic patterns for reference

171	[q11_v2]	Frage 10: Die Ableitung zeigt	radio, Required<br>1 Symmetrie in Amplitude und Frequenz, Theta-Aktivität intermittierende epilepsietypische Potenziale<br>2 Leichte Asymmetrie in Amplitude und Frequenz, Delta-Aktivität, Vigilanz beurteilen<br>888 Ausgeprägte Asymmetrie bezüglich Amplitude, Symmetrie hinsichtlich Frequenz, Zur Beurteilung ob echt: Elektroden testen.<br>5 Leichte Asymmetrie in Frequenz, Delta-Aktivität<br>3 Symmetrisch in Frequenz und Amplitude, mit EKG Artefakten, Alpha-Aktivität
172	[guess_q11_v2]	Bei der vorherigen Frage...	radio, Required<br>1 Ja - ich wusste die Antwort<br>2 Nein - ich habe geraten
173	[q10_poceeg_v2]	Section Header:<br>Frage 11: Falls Du das Bild grösser haben willst -> mit rechter Maustaste auf Bild klicken und "in neuem Tab öffnen" wählen	descriptive
174	[bsc_patterns10_v2]	EEG Grundrhythmen nachschauen (auf Link klicken)	descriptive<br>Field Annotation: basic patterns for reference
175	[q10_v2]	Frage 11: Die Ableitung zeigt	radio, Required<br>1 Symmetrie in Amplitude und Frequenz, Theta-Aktivität, epilepsietypische Potenziale<br>2 Leichte Asymmetrie in Amplitude, Beta-Aktivität<br>4 Ausgeprägte Asymmetrie in Amplitude, Alpha-Aktivität<br>888 Symmetrie in Amplitude und Frequenz, Delta-Theta Aktivität, Kind schläft wahrscheinlich (klinisch schauen)<br>3 Ausgeprägte Asymmetrie, epileptische Anfallsmuster auf Ableitung 2
176	[guess_q10_v2]	Bei der vorherigen Frage...	radio, Required<br>1 Ja - ich wusste die Antwort<br>2 Nein - ich habe geraten
177	[q12_poceeg_v2]	Section Header:<br>Frage 12:	descriptive
178	[bsc_patterns12_v2]	EEG Grundrhythmen nachschauen (auf Link klicken)	descriptive<br>Field Annotation: basic patterns for reference
179	[q12_v2]	Frage 12: Die Ableitung zeigt	radio, Required<br>1 Symmetrie in Amplitude und Frequenz, vereinzelte epilepsietypische Potenziale<br>2 Ausgeprägte Asymmetrie in Amplitude, symmetrische Frequenz, vorwiegend Beta-Aktivität<br>888 Symmetrisch in Amplitude und Frequenz, vorwiegend Delta-Theta Aktivität<br>5 leichte Asymmetrie in Frequenz, Beta Aktivität<br>3 Symmetrie Amplitude und Frequenz, Bewegungsartefakte
180	[guess_q12_v2]	Bei der vorherigen Frage...	radio, Required<br>1 Ja - ich wusste die Antwort<br>2 Nein - ich habe geraten
181	[bsc_knwl_v2]	Section Header: *post-Test Ende*<br>Correct Basic knowledge	calc<br>Calculation: sum([basic_knw_1_v2],[basic_knw_2_v2],[basic_knw_3_v2],[basic_knw_4_v2],[basic_knw_5_v2])<br>Field Annotation: @HIDDEN

182	[total_guessrte_v2]	Total guessrate (nein ich habe geraten) out of 12	calc<br>Calculation: sum([gsrte1_v2],[gsrte2_v2],[gsrte3_v2],[gsrte4_v2],[gsrte5_v2],[gsrte6_v2],[gsrte7_v2],[gsrte8_v2],[gsrte9_v2],[gsrte10_v2],[gsrte11_v2],[gsrte12_v2])<br>Field Annotation: @HIDDEN
183	[total_ngss_corr_v2]	Total no guess&correct	calc<br>Calculation: sum([nogss_corr_1_v2],[nogss_corr_2_v2],[nogss_corr_3_v2],[nogss_corr_4_v2],[nogss_corr_5_v2],[nogss_corr_5_v2],[nogss_corr_6_v2],[nogss_corr_7_v2],[nogss_corr_8_v2],[nogss_corr_9_v2],[nogss_corr_10_v2],[nogss_corr_11_v2],[nogss_corr_12_v2])<br>Field Annotation: @HIDDEN
184	[eeg_total_correct_v2]	Total correct out of 12	calc<br>Calculation: sum([q1_corr_v2],[q2_corr_v2],[q3_corr_v2],[q4_corr_v2],[q5_corr_v2],[q6_corr_v2],[q7_corr_v2],[q8_corr_v2],[q9_corr_v2],[q10_corr_v2],[q11_corr_v2],[q12_corr_v2])<br>Field Annotation: @HIDDEN
185	[eeg_artif_corr_v2]	Korrekte Artefakt pocEEG out of 4	calc<br>Calculation: sum([q1_corr_v2],[q2_corr_v2],[q8_corr_v2],[q11_corr_v2])<br>Field Annotation: @HIDDEN
186	[eeg_path_corr_v2]	Korrekte pathologische pocEEG out of 4	calc<br>Calculation: sum([q3_corr_v2],[q4_corr_v2],[q5_corr_v2],[q7_corr_v2])<br>Field Annotation: @HIDDEN
187	[eeg_norm_corr_v2]	Korrekte normale pocEEG out of 4	calc<br>Calculation: sum([q6_corr_v2],[q9_corr_v2],[q10_corr_v2],[q12_corr_v2])<br>Field Annotation: @HIDDEN
188	[gsrte1_v2]	Guess rate out of 1	calc<br>Calculation: if ([guess_q1_v2] = 2,1,0)<br>Field Annotation: @HIDDEN
189	[gsrte2_v2]	Guess rate 2	calc<br>Calculation: if ([guess_q2_v2] = 2,1,0)<br>Field Annotation: @HIDDEN
190	[gsrte3_v2]	Guess rate 3	calc<br>Calculation: if ([guess_q3_v2] = 2,1,0)<br>Field Annotation: @HIDDEN
191	[gsrte4_v2]	Guess rate 4	calc<br>Calculation: if ([guess_q4_v2] = 2,1,0)<br>Field Annotation: @HIDDEN
192	[gsrte5_v2]	Guess rate 5	calc<br>Calculation: if ([guess_q5_v2] = 2,1,0)<br>Field Annotation: @HIDDEN
193	[gsrte6_v2]	Guess rate 6	calc<br>Calculation: if ([guess_q6_v2] = 2,1,0)<br>Field Annotation: @HIDDEN
194	[gsrte7_v2]	Guess rate 7	calc<br>Calculation: if ([guess_q7_v2] = 2,1,0)<br>Field Annotation: @HIDDEN
195	[gsrte8_v2]	Guess rate 8	calc<br>Calculation: if ([guess_q8_v2] = 2,1,0)<br>Field Annotation: @HIDDEN
196	[gsrte9_v2]	Guess rate 9	calc<br>Calculation: if ([guess_q9_v2] = 2,1,0)<br>Field Annotation: @HIDDEN
197	[gsrte10_v2]	Guess rate 10	calc<br>Calculation: if ([guess_q10_v2] = 2,1,0)<br>Field Annotation: @HIDDEN
198	[gsrte11_v2]	Guess rate 11	calc<br>Calculation: if ([guess_q11_v2] = 2,1,0)<br>Field Annotation: @HIDDEN
199	[gsrte12_v2]	Guess rate 12	calc<br>Calculation: if ([guess_q12_v2] = 2,1,0)<br>Field Annotation: @HIDDEN

200	[q1_corr_v2]	q1 correct answer	calc<br>Calculation: if([q1_v2] = 888, 1, 0)<br>Field Annotation: @HIDDEN
201	[q2_corr_v2]	q2 correct answer	calc<br>Calculation: if([q2_v2] = 888, 1, 0)<br>Field Annotation: @HIDDEN
202	[q3_corr_v2]	q3 correct answer	calc<br>Calculation: if([q3_v2] = 888, 1, 0)<br>Field Annotation: @HIDDEN
203	[q4_corr_v2]	q4 correct answer	calc<br>Calculation: if([q4_v2] = 888, 1, 0)<br>Field Annotation: @HIDDEN
204	[q5_corr_v2]	q5 correct answer	calc<br>Calculation: if([q5_v2] = 888, 1, 0)<br>Field Annotation: @HIDDEN
205	[q6_corr_v2]	q6 correct answer	calc<br>Calculation: if([q6_v2] = 888, 1, 0)<br>Field Annotation: @HIDDEN
206	[q7_corr_v2]	q7 correct answer	calc<br>Calculation: if([q7_v2] = 888, 1, 0)<br>Field Annotation: @HIDDEN
207	[q8_corr_v2]	q8 correct answer	calc<br>Calculation: if([q8_v2] = 888, 1, 0)<br>Field Annotation: @HIDDEN
208	[q9_corr_v2]	q9 correct answer	calc<br>Calculation: if([q9_v2] = 888, 1, 0)<br>Field Annotation: @HIDDEN
209	[q10_corr_v2]	q10 correct answer	calc<br>Calculation: if([q10_v2] = 888, 1, 0)<br>Field Annotation: @HIDDEN
210	[q11_corr_v2]	q11 correct answer	calc<br>Calculation: if([q11_v2] = 888, 1, 0)<br>Field Annotation: @HIDDEN
211	[q12_corr_v2]	q12 correct answer	calc<br>Calculation: if([q12_v2] = 888, 1, 0)<br>Field Annotation: @HIDDEN
212	[basic_knw_1_v2]	q sy correct answer	calc<br>Calculation: if([sy_v2] = 888, 1, 0)<br>Field Annotation: @HIDDEN
213	[basic_knw_2_v2]	q lat correct answer	calc<br>Calculation: if([lat_v2] = 888, 1, 0)<br>Field Annotation: @HIDDEN
214	[basic_knw_3_v2]	q t_1020 correct answer	calc<br>Calculation: if([t_1020_v2] = 888, 1, 0)<br>Field Annotation: @HIDDEN
215	[basic_knw_4_v2]	q f_1020 correct answer	calc<br>Calculation: if([f_1020_v2] = 888, 1, 0)<br>Field Annotation: @HIDDEN
216	[basic_knw_5_v2]	q t_1020 correct answer	calc<br>Calculation: if([knwl_amp_v2] = 888, 1, 0)<br>Field Annotation: @HIDDEN
217	[nogss_corr_1_v2]	no_guess_corr_q1	calc<br>Calculation: if([q1_v2] = 888 AND [guess_q1_v2] = 1, 1, 0)<br>Field Annotation: @HIDDEN
218	[nogss_corr_2_v2]	no_guess_corr_q2	calc<br>Calculation: if([q2_v2] = 888 AND [guess_q2_v2] = 1, 1, 0)<br>Field Annotation: @HIDDEN
219	[nogss_corr_3_v2]	no_guess_corr_q3	calc<br>Calculation: if([q3_v2] = 888 AND [guess_q3_v2] = 1, 1, 0)<br>Field Annotation: @HIDDEN
220	[nogss_corr_4_v2]	no_guess_corr_q4	calc<br>Calculation: if([q4_v2] = 888 AND [guess_q4_v2] = 1, 1, 0)<br>Field Annotation: @HIDDEN

221	[nogss_corr_5_v2]	no_guess_corr_q5	calc<br>Calculation: if([q5_v2] = 888 AND [guess_q5_v2] = 1, 1, 0)<br>Field Annotation: @HIDDEN
222	[nogss_corr_6_v2]	no_guess_corr_q6	calc<br>Calculation: if([q5_v2] = 888 AND [guess_q5_v2] = 1, 1, 0)<br>Field Annotation: @HIDDEN
223	[nogss_corr_7_v2]	no_guess_corr_q7	calc<br>Calculation: if([q7_v2] = 888 AND [guess_q7_v2] = 1, 1, 0)<br>Field Annotation: @HIDDEN
224	[nogss_corr_8_v2]	no_guess_corr_q8	calc<br>Calculation: if([q8_v2] = 888 AND [guess_q8_v2] = 1, 1, 0)<br>Field Annotation: @HIDDEN
225	[nogss_corr_9_v2]	no_guess_corr_q9	calc<br>Calculation: if([q9_v2] = 888 AND [guess_q9_v2] = 1, 1, 0)<br>Field Annotation: @HIDDEN
226	[nogss_corr_10_v2]	no_guess_corr_q10	calc<br>Calculation: if([q10_v2] = 888 AND [guess_q10_v2] = 1, 1, 0)<br>Field Annotation: @HIDDEN
227	[nogss_corr_11_v2]	no_guess_corr_q11	calc<br>Calculation: if([q11_v2] = 888 AND [guess_q11_v2] = 1, 1, 0)<br>Field Annotation: @HIDDEN
228	[nogss_corr_12_v2]	no_guess_corr_q12	calc<br>Calculation: if([q12_v2] = 888 AND [guess_q12_v2] = 1, 1, 0)<br>Field Annotation: @HIDDEN
229	[poceeg_posttest_complete]	Section Header: *Form Status*<br>Complete?	dropdown<br>0 Incomplete<br>1 Unverified<br>2 Complete
**Instrument: pocEEG Knowledge Retention Test** (poceeg_knowledge_retention_test)			**Enabled as survey**
230	[vorwort_v3]	Liebes Notfall-Team, Willkommen zum pocEEG - Lernmodul "Post-Test"Dieser Test dient der Auswertung wieviel Wissen ihr behalten konntet und es ist der letzte Test. Vielen Dank, dass ihr euch nochmals Zeit nehmt um die Fragen zu beantworten.	descriptive
231	[y_n_module_2]	Section Header:<br>Ich habe das Lernmodul gemacht	radio<br>1 ja<br>2 nein
232	[cptc_appl_v3]	Section Header:<br>Ich fühle mich kompetent ein pocEEG anzulegen bzw. die Anlage zu kontrollieren	radio (Matrix), Required<br>1 Stimme völlig zu<br>2 Stimme zu<br>3 Stimme weder zu noch nicht zu<br>4 Stimme nicht zu<br>5 Stimme überhaupt nicht zu
233	[cptc_sngl_v3]	Ich fühle mich kompetent ein pocEEG Signal auf dem Monitor erkennen	radio (Matrix), Required<br>1 Stimme völlig zu<br>2 Stimme zu<br>3 Stimme weder zu noch nicht zu<br>4 Stimme nicht zu<br>5 Stimme überhaupt nicht zu
234	[cptc_intrp_v3]	Ich fühle mich kompetent ein pocEEG zu interpretieren z.B. Status epilepticus	radio (Matrix), Required<br>1 Stimme völlig zu<br>2 Stimme zu<br>3 Stimme weder zu noch nicht zu<br>4 Stimme nicht zu<br>5 Stimme überhaupt nicht zu

235	[cptc_art_v3]	Ich weiss welche Artefakte auftreten können	radio (Matrix), Required<br>1 Stimme völlig zu<br>2 Stimme zu<br>3 Stimme weder zu noch nicht zu<br>4 Stimme nicht zu<br>5 Stimme überhaupt nicht zu
236	[cptc_art_ex_v3]	Ich weiss wie ich Artefakte beheben kann	radio (Matrix), Required<br>1 Stimme völlig zu<br>2 Stimme zu<br>3 Stimme weder zu noch nicht zu<br>4 Stimme nicht zu<br>5 Stimme überhaupt nicht zu
237	[utlty_v3]	Aus jetziger Sicht: wie schätzt du die Nützlichkeit eines pocEEG auf der Notfallstation ein?	radio, Required<br>1 Äusserst hilfreich<br>2 Sehr hilfreich<br>3 Etwas hilfreich<br>4 Nur bedingt hilfreich<br>5 Überhaupt nicht hilfreich
238	[sy_v3]	Section Header:<br>Was ist das internationale 10-20 System?	radio, Required<br>1 Es wurde zwischen 1910 und 1920 erfunden<br>2 Ableitungen mit 10 bis 20 Kanälen<br>888 Standardisierte Elektroden-Platzierung<br>4 Man benötigt nur 10 bis 20 Elektroden
239	[lat_v3]	Section Header:<br>Gemäss 10-20 System für EEG-Ableitung, sind GERADE Zahlen (=2,4,6,...) welcher Seite des Gehirns zugeordnet?	radio, Required<br>1 Links<br>2 Frontal<br>888 Rechts<br>4 Temporal
240	[t_1020_v3]	Was bedeutet Buchstabe T im 10-20 System?	radio, Required<br>888 Temporallappen<br>2 Thalamus<br>3 Tectum<br>4 Trigeminus
241	[f_1020_v3]	Section Header:<br>Was bedeutet Buchstabe F im 10-20 System?	radio, Required<br>1 Facialis<br>888 Frontallappen<br>3 Falx<br>4 Fornix
242	[knwl_amp_v3]	Welche Einheit beschreibt die Amplitude des EEG (die Höhe der Welle)?	radio, Required<br>1 Univolt<br>2 Milliampere (mA)<br>888 Mikrovolt (µV)<br>4 Hertz (Hz)<br>5 Kilowatt (kW)
243	[mc_instr_3]	Section Header:<br>Im nächsten Abschnitt kommen Fragen zur pocEEG Befunden.Grafik kann wie bei jedem Bild beschrieben bei Bedarf vergrössert werden, Videos ebenfalls via Symbol direkt im Videofeld.Es nur eine der fünf Antworten ist richtig.Hinweis: Achtet bei der Beantwortung auf stimmige Symmetrie UND Grundrhythmus des EEG (letztere sind bei Bedarf bei jeder Frage via Link einsehbar).	descriptive
244	[q5_poceeg_v3]	Section Header: *pocEEG Befunde*<br>Frage 1	descriptive
245	[bsc_patterns5_v3]	EEG Grundrhythmen nachschauen (auf Link klicken)	descriptive<br>Field Annotation: basic patterns for reference

246	[q5_v3]	Frage 1: Die Ableitung zeigt	radio, Required<br>1 Symmetrie in Amplitude und Frequenz, Beta-Aktivität Ableitung 1<br>2 Eingelagerte Artefakte von Augenbewegungen<br>888 Anfallsmuster auf beiden Ableitungen<br>4 Leichte Asymmetrie in Amplitude, vorwiegend Theta-Aktivität<br>5 Ausgeprägte Asymmetrie in Frequenz, vorwiegend Beta-Aktivität
247	[guess_q5_v3]	Bei der vorherigen Frage...	radio, Required<br>1 Ja - ich wusste die Antwort<br>2 Nein - ich habe geraten
248	[q12_poceeg_v3]	Section Header:<br>Frage 2:	descriptive
249	[bsc_patterns12_v3]	EEG Grundrhythmen nachschauen (auf Link klicken)	descriptive<br>Field Annotation: basic patterns for reference
250	[q12_v3]	Frage 2: Die Ableitung zeigt	radio, Required<br>1 Symmetrie in Amplitude und Frequenz, vereinzelte epilepsietypische Potenziale<br>2 Ausgeprägte Asymmetrie in Amplitude, symmetrische Frequenz, vorwiegend Beta-Aktivität<br>888 Symmetrisch in Amplitude und Frequenz, vorwiegend Delta-Theta Aktivität<br>5 leichte Asymmetrie in Frequenz, Beta Aktivität<br>3 Symmetrie Amplitude und Frequenz, Bewegungsartefakte
251	[guess_q12_v3]	Bei der vorherigen Frage...	radio, Required<br>1 Ja - ich wusste die Antwort<br>2 Nein - ich habe geraten
252	[q11_poceeg_v3]	Section Header:<br>Frage 3:<br><br>Falls Du das Bild grösser haben willst -> mit rechter Maustaste auf Bild klicken und "in neuem Tab öffnen" wählen	descriptive
253	[bsc_patterns11_v3]	EEG Grundrhythmen nachschauen (auf Link klicken)	descriptive<br>Field Annotation: basic patterns for reference
254	[q11_v3]	Frage 3: Die Ableitung zeigt	radio, Required<br>1 Symmetrie in Amplitude und Frequenz, Theta-Aktivität intermittierende epilepsietypische Potenziale<br>2 Leichte Asymmetrie in Amplitude und Frequenz, Delta-Aktivität, Vigilanz beurteilen<br>888 Ausgeprägte Asymmetrie bezüglich Amplitude, Symmetrie hinsichtlich Frequenz, Zur Beurteilung ob echt: Elektroden testen.<br>5 Leichte Asymmetrie in Frequenz, Delta-Aktivität<br>3 Symmetrisch in Frequenz und Amplitude, mit EKG Artefakten, Alpha-Aktivität
255	[guess_q11_v3]	Bei der vorherigen Frage...	radio, Required<br>1 Ja - ich wusste die Antwort<br>2 Nein - ich habe geraten
256	[q10_poceeg_v3]	Section Header:<br>Frage 4: Falls Du das Bild grösser haben willst -> mit rechter Maustaste auf Bild klicken und "in neuem Tab öffnen" wählen	descriptive
257	[bsc_patterns10_v3]	EEG Grundrhythmen nachschauen (auf Link klicken)	descriptive<br>Field Annotation: basic patterns for reference

258	[q10_v3]	Frage 4: Die Ableitung zeigt	radio, Required<br>1 Symmetrie in Amplitude und Frequenz, Theta-Aktivität, epilepsietypische Potenziale<br>2 Leichte Asymmetrie in Amplitude, Beta-Aktivität<br>4 Ausgeprägte Asymmetrie in Amplitude, Alpha-Aktivität<br>888 Symmetrie in Amplitude und Frequenz, Delta-Theta Aktivität, Kind schläft wahrscheinlich (klinisch schauen)<br>3 Ausgeprägte Asymmetrie, epileptische Anfallsmuster auf Ableitung 2
259	[guess_q10_v3]	Bei der vorherigen Frage...	radio, Required<br>1 Ja - ich wusste die Antwort<br>2 Nein - ich habe geraten
260	[q9_poceeg_v3]	Section Header:<br>Frage 5:	descriptive
261	[bsc_patterns9_v3]	EEG Grundrhythmen nachschauen (auf Link klicken)	descriptive<br>Field Annotation: basic patterns for reference
262	[q9_v3]	Frage 5: Die Ableitung zeigt	radio, Required<br>888 Symmetrie in Amplitude und Frequenz, vorwiegend Delta-Theta Mischaktivität, keine Anfallsmuster<br>2 Ausgeprägte Asymmetrie in Amplitude, Beta-Aktivität, St. n. Benzodiazepin-Gabe<br>4 Ausgeprägte Asymmetrie in Frequenz, Alpha-Aktivität<br>5 leichte Asymmetrie in Frequenz, Subdelta-Aktivität, epilepsietypische Potenziale<br>3 Epileptische Anfallsmuster auf Ableitung 1
263	[guess_q9_v3]	Bei der vorherigen Frage...	radio, Required<br>1 Ja - ich wusste die Antwort<br>2 Nein - ich habe geraten
264	[q8_poceeg_v3]	Section Header:<br>Frage 6:	descriptive
265	[bsc_patterns8_v3]	EEG Grundrhythmen nachschauen (auf Link klicken)	descriptive<br>Field Annotation: basic patterns for reference
266	[q8_v3]	Frage 6: Die Ableitung zeigt	radio, Required<br>1 Symmetrie in Amplitude und Frequenz, Alpha-Aktivität, intermittierende epilepsietypische Potenziale<br>2 Leichte Asymmetrie in Amplitude, vorwiegend Beta-Aktivität<br>4 Ausgeprägte Asymmetrie in Amplitude, Theta-Aktivität<br>5 Leichte Asymmetrie in Frequenz, Delta-Aktivität<br>888 Hochamplitudige Spitzen auf beiden Ableitungen- Epileptisch oder Muskelartefakt - Korrelation mit Klinik nötig
267	[guess_q8_v3]	Bei der vorherigen Frage...	radio, Required<br>1 Ja - ich wusste die Antwort<br>2 Nein - ich habe geraten
268	[q6_poceeg_v3]	Section Header:<br>Frage 7Falls Du das Bild (noch) grösser haben willst -> mit rechter Maustaste auf Bild klicken und "in neuem Tab öffnen" wählen	descriptive
269	[bsc_patterns6_v3]	EEG Grundrhythmen nachschauen (auf Link klicken)	descriptive<br>Field Annotation: basic patterns for reference

270	[q6_v3]	Frage 7: Die Ableitung zeigt	radio, Required<br>888 Symmetrisch, vorwiegende Delta-Aktivität, normales Schlaf-EEG.<br>2 Leichte Asymmetrie in Amplitude, Beta-Aktivität<br>4 Ausgeprägte Asymmetrie in Amplitude, Theta-Aktivität<br>5 Leichte Asymmetrie in Frequenz, Delta-Aktivität<br>3 Epileptische Anfallsmuster auf beiden Ableitungen
271	[guess_q6_v3]	Bei der vorherigen Frage...	radio, Required<br>1 Ja - ich wusste die Antwort<br>2 Nein - ich habe geraten
272	[q4_poceeg_v3]	Section Header:<br>Frage 8Falls Du das Bild grösser haben willst -> mit rechter Maustaste auf Bild klicken und "in neuem Tab öffnen" wählen	descriptive
273	[bsc_patterns4_v3]	EEG Grundrhythmen nachschauen (auf Link klicken)	descriptive<br>Field Annotation: basic patterns for reference
274	[q4_v3]	Frage 8: Die Ableitung auf dem Bild zeigt	radio, Required<br>1 Störartefakt von technischem Gerät<br>2 Symmetrisch in Frequenz und Amplitude, normale Delta-Aktivität<br>3 Leichte Asymmetrie in Amplitude, vorwiegend Beta-Aktivität<br>888 Deutliche Asymmetrie in Amplitude, epileptische Anfallsmuster<br>5 Normale kortikale Aktivität
275	[guess_q4_v3]	Bei der vorherigen Frage...	radio, Required<br>1 Ja - ich wusste die Antwort<br>2 Nein - ich habe geraten
276	[q3_poceeg_v3]	Section Header:<br>Frage 9Falls Du das Bild grösser haben willst -> mit rechter Maustaste auf Bild klicken und "in neuem Tab öffnen" wählen	descriptive
277	[bsc_patterns3_v3]	EEG Grundrhythmen nachschauen (auf Link klicken)	descriptive<br>Field Annotation: basic patterns for reference
278	[q3_v3]	Frage 9: Die Ableitung zeigt	radio, Required<br>1 Symmetrie in Amplitude und Frequenz, Beta-Aktivität Ableitung 1<br>2 Eingelagerte Artefakte von Augenbewegungen<br>3 Epileptische Anfallsmuster auf beiden Ableitungen<br>4 Asymmetrie in Amplitude, Delta-Aktivität Ableitung 2<br>888 Asymmetrie in Frequenz und Amplitude, Beta Aktivität-Ableitung 2
279	[guess_q3_v3]	Bei der vorherigen Frage...	radio, Required<br>1 Ja - ich wusste die Antwort<br>2 Nein - ich habe geraten
280	[q2_poceeg_v3]	Section Header:<br>Frage 10Falls Du das Bild grösser haben willst -> mit rechter Maustaste auf Bild klicken und "in neuem Tab öffnen" wählen	descriptive
281	[bsc_patterns2_v3]	EEG Grundrhythmen nachschauen (auf Link klicken)	descriptive<br>Field Annotation: basic patterns for reference

282	[q2_v3]	Frage 10: Die Ableitung zeigt	radio, Required<br>1 Ausgeprägte Asymmetrie in Amplitude und Frequenz, Elektrodenartefakte<br>888 Eingelagerte Artefakte von Augenbewegungen<br>3 Epileptische Anfallsmuster auf beiden Ableitungen<br>4 Asymmetrie in Amplitude, Theta-Aktivität<br>5 EKG Artefakte
283	[guess_q2_v3]	Bei der vorherigen Frage...	radio, Required<br>1 Ja - ich wusste die Antwort<br>2 Nein - ich habe geraten
284	[q1_poceeg_v3]	Section Header:<br>Frage 11Falls Du das Bild grösser haben willst -> mit rechter Maustaste auf Bild klicken und "in neuem Tab öffnen" wählen	descriptive
285	[bsc_patterns1_v3]	EEG Grundrhythmen nachschauen (auf Link klicken)	descriptive<br>Field Annotation: basic patterns for reference
286	[q1_v3]	Frage 11: Die Ableitung zeigt	radio, Required<br>1 Symmetrie in Amplitude und Frequenz, Beta-Aktivität<br>2 Eingelagerte Artefakte von Muskelaktivität<br>3 Epileptische Entladungen auf beiden Ableitungen<br>4 Ausgeprägte Asymmetrie in Amplitude, Theta-Aktivität<br>888 Symmetrisch, Delta-Aktivität, EKG Artefakte
287	[guess_q1_v3]	Bei der vorherigen Frage...	radio, Required<br>1 Ja - ich wusste die Antwort<br>2 Nein - ich habe geraten
288	[q7_poceeeg_v3]	Section Header:<br>Frage 12:	descriptive
289	[bsc_patterns7_v3]	EEG Grundrhythmen nachschauen (auf Link klicken)	descriptive<br>Field Annotation: basic patterns for reference
290	[q7_v3]	Frage 12: Die Ableitung zeigt	radio, Required<br>1 Weitgehende Symmetrie in Amplitude und Frequenz, Theta-Delta Aktivität, vereinzelt epilepsietypische Potenziale<br>2 Ausgeprägte Asymmetrie in Amplitude und Frequenz, Beta-Aktivität<br>888 Epileptische Anfallsmuster in beiden Ableitungen<br>4 Ausgeprägte Asymmetrie in Amplitude, vorwiegend Alpha-Aktivität<br>5 leichte Asymmetrie in Frequenz, vorwiegend Beta-Aktivität
291	[guess_q7_v3]	Bei der vorherigen Frage...	radio, Required<br>1 Ja - ich wusste die Antwort<br>2 Nein - ich habe geraten
292	[bsc_knwl_v3]	Section Header: *Ende Test*<br>Correct Basic Knowledge 3mt	calc<br>Calculation: sum([basic_knw_1_v3],[basic_knw_2_v3],[basic_knw_3_v3],[basic_knw_4_v3],[basic_knw_5_v3])<br>Field Annotation: @HIDDEN
293	[total_guessrte_v3]	Total guessrate (nein ich habe geraten) out of 12	calc<br>Calculation: sum([gsrte1_v3],[gsrte2_v3],[gsrte3_v3],[gsrte4_v3],[gsrte5_v3],[gsrte6_v3],[gsrte7_v3],[gsrte8_v3],[gsrte9_v3],[gsrte10_v3],[gsrte11_v3],[gsrte12_v3])<br>Field Annotation: @HIDDEN

294	[total_ngss_corr_v3]	Total no guess&correct	calc<br>Calculation: sum([nogss_corr_1_v3],[nogss_corr_2_v3],[nogss_corr_3_v3],[nogss_corr_4_v3],[nogss_corr_5_v3],[nogss_corr_5_v3],[nogss_corr_6_v3],[nogss_corr_7_v3],[nogss_corr_8_v3],[nogss_corr_9_v3],[nogss_corr_10_v3],[nogss_corr_11_v3],[nogss_corr_12_v3])<br>Field Annotation: @HIDDEN
295	[eeg_total_correct_v3]	Total correct out of 12	calc<br>Calculation: sum([q1_corr_v3],[q2_corr_v3],[q3_corr_v3],[q4_corr_v3],[q5_corr_v3],[q6_corr_v3],[q7_corr_v3],[q8_corr_v3],[q9_corr_v3],[q10_corr_v3],[q11_corr_v3],[q12_corr_v3])<br>Field Annotation: @HIDDEN
296	[eeg_artif_corr_v3]	Korrekte Artefakt pocEEG out of 4	calc<br>Calculation: sum([q1_corr_v3],[q2_corr_v3],[q8_corr_v3],[q11_corr_v3])<br>Field Annotation: @HIDDEN Artefakte q1-2-8-11
297	[eeg_path_corr_v3]	Korrekte pathologische pocEEG out of 4	calc<br>Calculation: sum([q3_corr_v3],[q4_corr_v3],[q5_corr_v3],[q7_corr_v3])<br>Field Annotation: @HIDDEN
298	[eeg_norm_corr_v3]	Korrekte normale pocEEG out of 4	calc<br>Calculation: sum([q6_corr_v3],[q9_corr_v3],[q10_corr_v3],[q12_corr_v3])<br>Field Annotation: @HIDDEN
299	[gsrte1_v3]	Guess rate out of 1	calc<br>Calculation: if ([guess_q1_v3] = 2,1,0)<br>Field Annotation: @HIDDEN
300	[gsrte2_v3]	Guess rate 2	calc<br>Calculation: if ([guess_q2_v3] = 2,1,0)<br>Field Annotation: @HIDDEN
301	[gsrte3_v3]	Guess rate 3	calc<br>Calculation: if ([guess_q3_v3] = 2,1,0)<br>Field Annotation: @HIDDEN
302	[gsrte4_v3]	Guess rate 4	calc<br>Calculation: if ([guess_q4_v3] = 2,1,0)<br>Field Annotation: @HIDDEN
303	[gsrte5_v3]	Guess rate 5	calc<br>Calculation: if ([guess_q5_v3] = 2,1,0)<br>Field Annotation: @HIDDEN
304	[gsrte6_v3]	Guess rate 6	calc<br>Calculation: if ([guess_q6_v3] = 2,1,0)<br>Field Annotation: @HIDDEN
305	[gsrte7_v3]	Guess rate 7	calc<br>Calculation: if ([guess_q7_v3] = 2,1,0)<br>Field Annotation: @HIDDEN
306	[gsrte8_v3]	Guess rate 8	calc<br>Calculation: if ([guess_q8_v3] = 2,1,0)<br>Field Annotation: @HIDDEN
307	[gsrte9_v3]	Guess rate 9	calc<br>Calculation: if ([guess_q9_v3] = 2,1,0)<br>Field Annotation: @HIDDEN
308	[gsrte10_v3]	Guess rate 10	calc<br>Calculation: if ([guess_q10_v3] = 2,1,0)<br>Field Annotation: @HIDDEN
309	[gsrte11_v3]	Guess rate 11	calc<br>Calculation: if ([guess_q11_v3] = 2,1,0)<br>Field Annotation: @HIDDEN
310	[gsrte12_v3]	Guess rate 12	calc<br>Calculation: if ([guess_q12_v3] = 2,1,0)<br>Field Annotation: @HIDDEN
311	[q1_corr_v3]	q1 correct answer	calc<br>Calculation: if([q1_v3] = 888, 1, 0)<br>Field Annotation: @HIDDEN
312	[q2_corr_v3]	q2 correct answer	calc<br>Calculation: if([q2_v3] = 888, 1, 0)<br>Field Annotation: @HIDDEN

313	[q3_corr_v3]	q3 correct answer	calc<br>Calculation: if([q3_v3] = 888, 1, 0)<br>Field Annotation: @HIDDEN
314	[q4_corr_v3]	q4 correct answer	calc<br>Calculation: if([q4_v3] = 888, 1, 0)<br>Field Annotation: @HIDDEN
315	[q5_corr_v3]	q5 correct answer	calc<br>Calculation: if([q5_v3] = 888, 1, 0)<br>Field Annotation: @HIDDEN
316	[q6_corr_v3]	q6 correct answer	calc<br>Calculation: if([q6_v3] = 888, 1, 0)<br>Field Annotation: @HIDDEN
317	[q7_corr_v3]	q7 correct answer	calc<br>Calculation: if([q7_v3] = 888, 1, 0)<br>Field Annotation: @HIDDEN
318	[q8_corr_v3]	q8 correct answer	calc<br>Calculation: if([q8_v3] = 888, 1, 0)<br>Field Annotation: @HIDDEN
319	[q9_corr_v3]	q9 correct answer	calc<br>Calculation: if([q9_v3] = 888, 1, 0)<br>Field Annotation: @HIDDEN
320	[q10_corr_v3]	q10 correct answer	calc<br>Calculation: if([q10_v3] = 888, 1, 0)<br>Field Annotation: @HIDDEN
321	[q11_corr_v3]	q11 correct answer	calc<br>Calculation: if([q11_v3] = 888, 1, 0)<br>Field Annotation: @HIDDEN
322	[q12_corr_v3]	q12 correct answer	calc<br>Calculation: if([q12_v3] = 888, 1, 0)<br>Field Annotation: @HIDDEN
323	[basic_knw_1_v3]	q sy correct answer	calc<br>Calculation: if([sy_v3] = 888, 1, 0)<br>Field Annotation: @HIDDEN
324	[basic_knw_2_v3]	q lat correct answer	calc<br>Calculation: if([lat_v3] = 888, 1, 0)<br>Field Annotation: @HIDDEN
325	[basic_knw_3_v3]	q t_1020 correct answer	calc<br>Calculation: if([t_1020_v3] = 888, 1, 0)<br>Field Annotation: @HIDDEN
326	[basic_knw_4_v3]	q f_1020 correct answer	calc<br>Calculation: if([f_1020_v3] = 888, 1, 0)<br>Field Annotation: @HIDDEN
327	[basic_knw_5_v3]	q t_1020 correct answer	calc<br>Calculation: if([knwl_amp_v3] = 888, 1, 0)<br>Field Annotation: @HIDDEN
328	[nogss_corr_1_v3]	no_guess_corr_q1	calc<br>Calculation: if([q1_v3] = 888 AND [guess_q1_v3] = 1, 1, 0)<br>Field Annotation: @HIDDEN
329	[nogss_corr_2_v3]	no_guess_corr_q2	calc<br>Calculation: if([q2_v3] = 888 AND [guess_q2_v3] = 1, 1, 0)<br>Field Annotation: @HIDDEN
330	[nogss_corr_3_v3]	no_guess_corr_q3	calc<br>Calculation: if([q3_v3] = 888 AND [guess_q3_v3] = 1, 1, 0)<br>Field Annotation: @HIDDEN
331	[nogss_corr_4_v3]	no_guess_corr_q4	calc<br>Calculation: if([q4_v3] = 888 AND [guess_q4_v3] = 1, 1, 0)<br>Field Annotation: @HIDDEN
332	[nogss_corr_5_v3]	no_guess_corr_q5	calc<br>Calculation: if([q5_v3] = 888 AND [guess_q5_v3] = 1, 1, 0)<br>Field Annotation: @HIDDEN
333	[nogss_corr_6_v3]	no_guess_corr_q6	calc<br>Calculation: if([q5_v3] = 888 AND [guess_q5_v3] = 1, 1, 0)<br>Field Annotation: @HIDDEN

334	[nogss_corr_7_v3]	no_guess_corr_q7	calc<br>Calculation: if([q7_v3] = 888 AND [guess_q7_v3] = 1, 1, 0)<br>Field Annotation: @HIDDEN
335	[nogss_corr_8_v3]	no_guess_corr_q8	calc<br>Calculation: if([q8_v3] = 888 AND [guess_q8_v3] = 1, 1, 0)<br>Field Annotation: @HIDDEN
336	[nogss_corr_9_v3]	no_guess_corr_q9	calc<br>Calculation: if([q9_v3] = 888 AND [guess_q9_v3] = 1, 1, 0)<br>Field Annotation: @HIDDEN
337	[nogss_corr_10_v3]	no_guess_corr_q10	calc<br>Calculation: if([q10_v3] = 888 AND [guess_q10_v3] = 1, 1, 0)<br>Field Annotation: @HIDDEN
338	[nogss_corr_11_v3]	no_guess_corr_q11	calc<br>Calculation: if([q11_v3] = 888 AND [guess_q11_v3] = 1, 1, 0)<br>Field Annotation: @HIDDEN
339	[nogss_corr_12_v3]	no_guess_corr_q12	calc<br>Calculation: if([q12_v3] = 888 AND [guess_q12_v3] = 1, 1, 0)<br>Field Annotation: @HIDDEN
340	[poceeg_knowledge_retention_test_complete]	Section Header: *Form Status*<br>Complete?	dropdown<br>0 Incomplete<br>1 Unverified<br>2 Complete
