# Supplementary material for: E-Learning for Pediatric Emergency Department Staff in Point-of-Care Electroencephalogram Interpretation: Prospective Cohort Study
Source: JMIR Med Educ. 2025 Aug 20;11:e69395. doi: 10.2196/69395 (PMC12370458; doi:10.2196/69395)
Supplement: Checklist 1 [file mededu-v11-e69395-s003.pdf]

Checklist for Reporting Results of Internet E-Surveys (CHERRIES)

Item Category	***Checklist Item***	***Explanation***
Design	Describe survey design	Longitudinal assessment with repeated measures over three test sessions (T1, T2, T3) to evaluate the effect of an EEG e-learning module.
IRB (Institutional Review Board) approval and informed consent process	IRB approval	Exempted by local IRB (Req-2024-00833)
		The subjects were informed of the purpose and anonymous nature of the test instruments (survey); informed consent was obtained electronically before participation.
	Data protection	The test was administered via REDCap, a secure, web-based application, hosted at the Children's Hospital Zurich, Switzerland. No identifiable information was stored with the survey results. Institutional email addresses of the participants were used to send invitations and reminders for participation in the respective test sessions. Record numbers were not linked to the email addresses.
Development and pretesting	Development and testing	The instrument was specifically developed for this study by subject experts. It underwent preliminary pilot testing by a pediatric neurology fellow not involved in the study to ensure clarity, feasibility and appropriate item difficulty.
Recruitment process and description of the sample having access to the questionnaire	Open survey versus closed survey	Closed survey - access was restricted to invited pediatric emergency staff at our institution
	Contact mode	Email invitations were sent through institutional channels.
	Advertising the survey	No public advertising was used; participants were recruited directly.

Survey administration	Web/E-mail	Web-based survey administered via REDCap.
	Context	Participants completed the instrument independently before (T1) and after (T2) interaction with the E-learning modiule, and at 3 months (T3) , outside clinical duties.
	Mandatory/voluntary	Participation was voluntary
	Incentives	No incentives were offered
	Time/Date	Data collection August 18, 2022 to March 6, 2023; Participants completed T1,T2,T3 at their convenience within predefined time window
	Randomization of items or questionnaires	Question order was randomized across the three test sessions to minimize bias..
	Adaptive questioning	No adaptive questioning; all participants received the same test items.
	Number of Items	Demographics, 5 basic EEG questions, 12 EEG interpretation tasks (7 static, 5 video) + confidence/self-assessment items (Likert).
	Number of screens (pages)	approx. 18 (depends on screen size, 8 page PDF)
	Completeness check	Participants could not proceed without answering each question.
	Review step	Participants could review and change answers before proceeding to the next question. Participants were not able to return to answered items..

Response rates	Unique site visitor	unique links; repeated entries prevented by REDCap .
	View rate (Ratio of uniquesurvey visitors/unique site vistors)	Not applicable as survey was by invitation only.

	Participation rate (Ratio of unique visitors who agreed to participate/unique first survey page visitors)	Calculated based on the number of invited vs. completed responses in T1, T2, and T3.
	Completion rate (Ratio of users who finished the survey/users who agreed to participate)	Calculated based on the number of participants in T1 vs. completed responses in T2 and T3.

Preventing multiple entries from the same individual	Cookies used	Not used; unique credentials for REDCap access
	IP check	Not used; unique credentials for REDCap access
	Log file analysis	N/A, repeated entries prevented by REDCap
	Registration	N/A

Analysis	Handling of incomplete questionnaires	Incomplete responses were excluded from analysis.
	Questionnaires submitted with an atypical timestamp	N/A. Personal invitation with reminders at predefined time windows round T1, T2 and T3.
	Statistical correction	No statistical corrections of the raw data were performed due to the focus on a specific pool of participants.

Adapted from Eysenbach G. Improving the quality of Web surveys: the Checklist for Reporting Results of Internet E-Surveys (CHERRIES). J Med Internet Res. 2004 Sep 29;6(3):e34. doi: 10.2196/jmir.6.3.e34
